# Advances in Polymer Micelles for Cancer Therapy: From Conventional to Smart Delivery Systems

**DOI:** 10.3390/pharmaceutics18020177

**Published:** 2026-01-29

**Authors:** Rayna Georgieva Bryaskova, Krasimir Georgiev Staykov, Damyan Stoyanov Ganchev

**Affiliations:** 1 Department of Polymer Engineering, University of Chemical Technology and Metallurgy, 1000 Sofia, Bulgaria; 2 Department of Machine Elements and Non-Metal Constructions, Technical University of Sofia, 1000 Sofia, Bulgaria; staikov@tu-sofia.bg (K.G.S.); ganchev_d@tu-sofia.bg (D.S.G.)

**Keywords:** nanomedicine, polymer micelles, drug release, chemotherapeutics, cancer therapy

## Abstract

Polymeric micelles have become a versatile and clinically significant class of nanocarriers for cancer therapy. They effectively solubilize poorly water-soluble anticancer drugs, extend their circulation in the bloodstream, and promote accumulation in tumors. Early studies focused on conventional PEG-based polymeric micelles that utilize passive targeting based on the enhanced permeability and retention (EPR) effect, with several of these advancing to clinical trials. Active targeting strategies using modified polymer micelles with various targeting ligands have been introduced to enhance cellular uptake and improve tumor specificity. Recently, the field has shifted toward smart polymer micelles that can respond to both internal (endogenous) and external (exogenous) stimuli. These stimuli-responsive systems enable controlled drug release, enhance delivery inside cells, and improve therapeutic effectiveness, all while reducing systemic toxicity. This review summarizes recent advancements in polymer design, drug-loading techniques, preparation methods, and targeting strategies for polymeric micelles, highlighting both preclinical progress and systems that have reached clinical stages. The transition from conventional to smart polymer micelles is a significant advancement toward achieving more precise, effective, and personalized cancer nanomedicine.

## 1. Introduction

Cancer continues to be one of the leading causes of illness and death worldwide, despite significant progress in diagnosis and treatment. According to the latest global estimates from the American Association for Cancer Research and the World Health Organization’s International Agency for Research on Cancer (IARC), approximately 20 million new cancer cases were diagnosed worldwide in 2022 [1,2]. This number is expected to remain similar or increase slightly by 2025 as global populations continue to age and grow. Each year, over 10 million people die from cancer, making it a major cause of death globally [2]. Current cancer treatments include surgical intervention, radiation, and chemotherapeutic drugs. Among them, conventional chemotherapy continues to play a central role in cancer treatment; however, its clinical effectiveness is often limited by the poor aqueous solubility of many anticancer drugs, unfavorable pharmacokinetics, nonspecific biodistribution, and severe systemic toxicities [3]. In particular, widely used classes of chemotherapeutic agents, such as taxanes, antracyclines, platinum-based drugs, and camptothecin derivatives, face formulation challenges and dose-limiting side effects, highlighting the urgent need for improved drug delivery strategies [4].

Nanotechnology-based drug delivery systems have emerged as a powerful approach to overcome the major limitations of conventional chemotherapy, such as poor solubility, rapid systemic clearance, nonspecific biodistribution, and dose-limiting toxicity. Various nanoscale carriers, including polymeric micelles [5,6], liposomes [7,8], polymeric nanoparticles [9], dendrimers [10], and inorganic nanocarriers [11], have been extensively investigated to improve drug solubility, enhance tumor accumulation via the enhanced permeability and retention (EPR) effect, and enable controlled or stimuli-responsive drug release. Among these systems, polymeric micelles formed from amphiphilic block copolymers are particularly attractive due to their small size, core–shell architecture, high drug-loading capacity for hydrophobic drugs, and ease of surface functionalization for active targeting. The hydrophobic core serves as a reservoir for poorly water-soluble anticancer agents, while the hydrophilic corona, often composed of poly(ethylene glycol) (PEG), provides steric stabilization, prolongs systemic circulation, and reduces recognition by the reticuloendothelial system [12]. Early generations of polymeric micelles primarily relied on passive targeting mechanisms, exploiting the EPR effect to preferentially accumulate in tumor tissues [13]. A number of micellar formulations based on passive targeting demonstrated improved pharmacokinetics, enhanced tumor accumulation, and reduced formulation-related toxicities compared to conventional solvent-based drugs, validating the clinical potential of polymeric micelles [14]. Despite these advancements, conventional polymeric micelles still encounter significant challenges, including premature drug leakage, limited tumor penetration, heterogeneous EPR effects among patients, and insufficient intracellular drug release [15]. One effective way to address these limitations is to design nanocarriers that can actively bind to specific cells after they have exited the bloodstream. This binding can be accomplished by attaching targeting agents, such as ligand molecules that specifically bind to receptors on the cell surface through ligand–receptor interactions and internalize into the cells, allowing the drug to be released inside the cell [11]. “Smart” polymer micellar systems with stimuli-responsive properties that allow for controlled drug release are the next-generation drug delivery systems. The drug release can be triggered by endogenous factors, which are intrinsic stimuli from the body’s unique pathways, or the characteristics of malignancies, as well as exogenous factors, which are external stimuli that enhance release [16]. Recent advances have further expanded the scope of polymeric micelles through multifunctional designs, including dual-ligand targeting, co-delivery of multiple drugs, and a combination of passive and active targeting strategies [17,18]. These developments represent a paradigm shift from simple drug solubilization platforms to intelligent nanocarriers capable of overcoming biological barriers and addressing tumor heterogeneity.

This review highlights recent progress in using polymeric micelles for cancer therapy, focusing on the transition from conventional PEG-based systems by passive and active targeting of cancer cells to novel, smart micelles that respond to stimuli and have specific targets depending on the applied stimuli. This review discusses key design principles of polymeric micelles, drug-loading strategies, and the main stimulus-responsive mechanisms, and summarizes representative preclinical and clinical studies.

## 2. Principles of Self-Assembly

The ability of a given molecule to self-organize and form aggregates with complex architecture depends on its amphiphilicity, which is determined by the presence of both hydrophobic and hydrophilic segments in its structure. The hydrophilic part can be anionically, cationically, or zwitterionically charged, or it may lack ionic functional groups altogether. In this way, the corresponding ionogenic or non-ionogenic amphiphilic molecules are formed [19]. Typical amphiphiles include various surfactants, as well as natural phospholipids and peptides [20,21]. In an aqueous environment, amphiphilic molecules can form particles that range in size from a nanometer to several micrometers, influenced by weak non-covalent interactions [22]. The thermodynamic driving force behind supramolecular organization primarily involves desolvation processes, conformational flexibility, and the intra- and intermolecular interactions between the hydrophobic segments of these molecules. Additionally, interaction forces from polar groups and hydrogen bonding can also affect the size and morphology of the resulting amphiphilic aggregates. Factors such as temperature, pH, and the ionic strength of the solvent have been shown to influence the characteristics of this supramolecular assembly [23]. Overall, the self-organization process is dynamic and can be controlled by modifying any of these parameters [24].

### 2.1. Self-Assembly of Amphiphilic Block Copolymers

Amphiphilic block copolymers that form micelles in aqueous systems have gained increasing attention in recent decades as drug delivery systems. They are designed to maximize the therapeutic effectiveness of drugs while minimizing their negative side effects. These copolymers consist of both hydrophobic and hydrophilic blocks that are covalently bonded together, functioning similarly to surfactants. Several methods are available for synthesizing amphiphilic block copolymers, including free radical polymerization (FRP) [25], ring-opening polymerization (ROP) [26,27,28], “living” anionic polymerization (LAP) [29], and controlled radical polymerization (CRP) [30]. Among these, controlled radical polymerizations are the most commonly used techniques, and include atom transfer radical polymerization (ATRP), nitroxide-mediated radical polymerization (NMP), and reversible addition–fragmentation chain transfer polymerization (RAFT) [30]. These approaches enable the synthesis of block copolymers with well-defined compositions, molecular weights, and complex architectures, which are able to self-assemble to a wide variety of well-defined structures in different morphologies [31,32,33,34].

**Figure 1:**
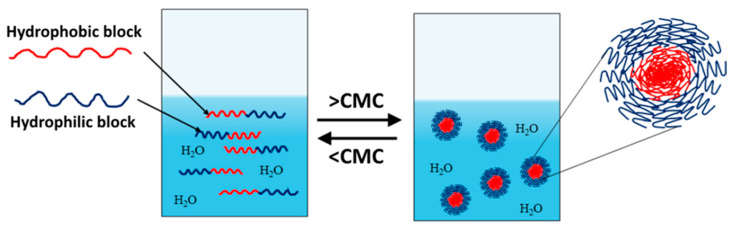
Unimer–micelle equilibrium of linear block copolymers in an aqueous environment.

Amphiphilic block copolymers dissolved in water as a selective solvent are the most commonly studied systems. Self-assembly is driven by the polymer chains’ need to minimize energetically unfavorable hydrophobic interactions. Therefore, micellization occurs as a result of two forces: the attractive forces between the hydrophobic blocks, which lead to the aggregation of the hydrophobic parts and the formation of the micelle core, and the repulsive forces between the hydrophilic segments, which form the micelle shell. The repulsive forces prevent unlimited micelle growth, while the interactions between the hydrophilic chains and the solvent further stabilize the micelles [32,33,34]. The micellization process of amphiphilic copolymers begins with the formation of a true solution, in which macromolecules exist in an unassociated state (unimers). Upon increasing the concentration and reaching the so-called critical micelle concentration (CMC), polymer micelles are formed in the system (Figure 1). At constant temperature, the resulting polymer particles exist in thermodynamic equilibrium with the unimers. This process is analogous to that of low-molecular-weight surfactants, with the difference being that amphiphilic block copolymers have much lower CMC values, making them stable and robust nanoscale systems [35].

**Figure 2:**
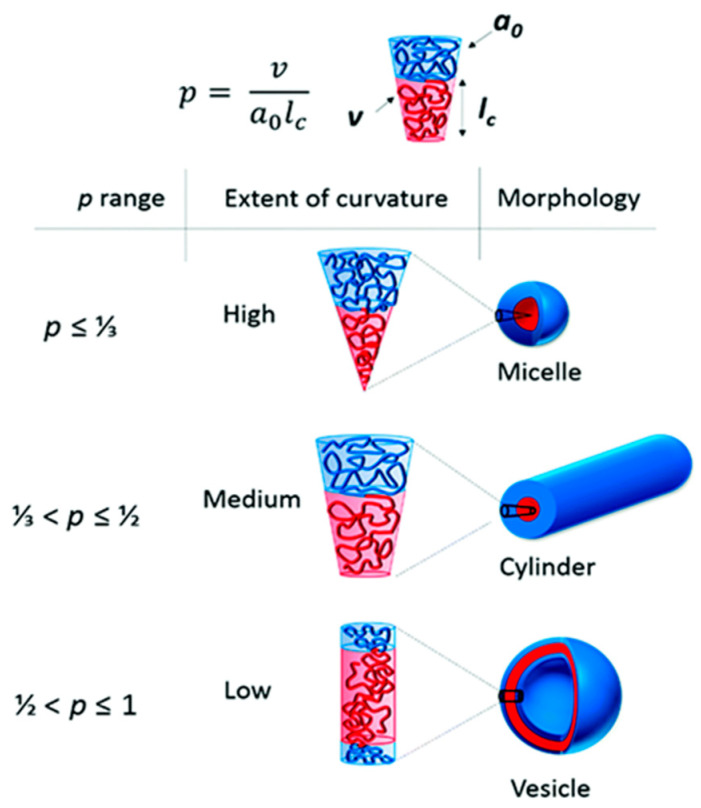
The different morphologies obtained depending on the packing parameters, *p* [36] (this article is licensed under a Creative Commons Attribution 3.0 Unported License).

Amphiphilic block copolymers, including diblock copolymers (AB type), triblock copolymers (ABA or ABC type), and multiblock copolymers, as well as double-hydrophilic copolymers, can self-assemble in an aqueous environment. This self-assembly leads to the formation of various nanostructures, such as spherical, “flower-like,” “worm-like,” and vesicular shapes. Several factors influence the self-assembling behavior of these polymers, including temperature, pH, salt concentration, polymer concentration, the type of solvent used, and the structure and length of the polymer blocks [31]. The formation of polymer micelles with different morphologies can be predicted based on the so-called packing parameter *p* = ν/(a_0_.l_c_). This parameter is a key characteristic of amphiphilic polymers and is determined by their composition, where v is the volume of the hydrophobic chain, a_0_ is the optimal area of the head group, and lc is the length of the hydrophobic tail (Figure 2). As a rule, spherical micelles are favored when *p* ≤ 1/3, cylindrical micelles are favored when 1/3 ≤ *p* ≤ 1/2, and enclosed membrane structures (vesicles, also known as polymersomes) are favored when 1/2 ≤ *p* ≤ 1 [36].

### 2.2. Methods for the Preparation of Polymer Micelles for Drug Delivery

**Figure 3:**
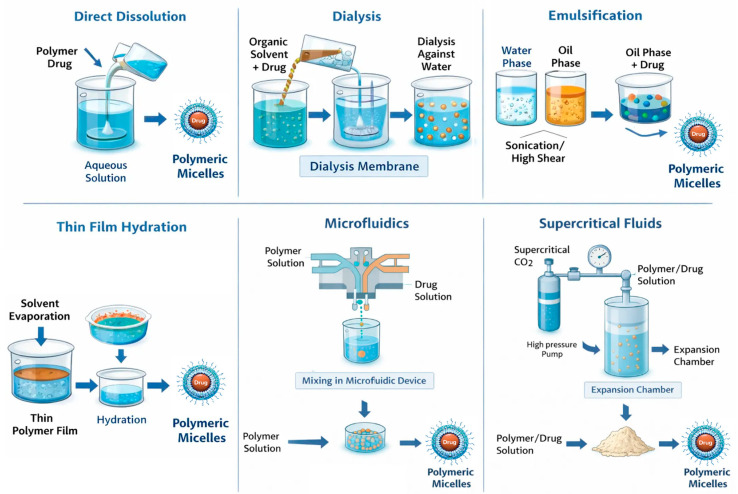
Polymer micelle preparation strategies.

The method used to prepare polymer micelles is based on the physicochemical properties of the selected block copolymers. Importantly, this method significantly impacts the physicochemical parameters and drug encapsulation efficiency (Figure 3). Additionally, factors such as the order of addition, the ratio of aqueous to organic phases, and the concentration of copolymers affect the size, polydispersity index, and stability of the resulting micelles [37,38,39]. The preparation of polymer micelles for drug delivery is primarily carried out using two main methods [32,38]. The first method is a direct dissolution that involves a step of dissolving the block copolymer and drug in a suitable solvent. The micelle formation process occurs after thermal and/or ultrasonic treatment. This approach is mainly applicable to copolymers with low molecular weight and relatively short insoluble blocks. Depending on the type of block copolymer, equilibrium may not always be reached in the system, especially when the block forming the core has a high glass transition temperature. In such cases, so-called “frozen” micelles are formed, meaning that no exchange occurs between the micelles and the unimers [32]. The second method for preparing polymer micelles is a solvent exchange and involves processes that alter the solubility of the amphiphilic block copolymer. To achieve this, the block copolymer and the drug are first dissolved in a “good” solvent for both blocks (forming a real, true solution), and then the solvent composition is altered by gradually adding a second, “poor” solvent. The added second solvent is suitable for only one of the blocks. Gradual replacement of the common solvent with a selective one can also be achieved through evaporation of the initial solvent or by dialysis. In this way, micellar systems in aqueous environments are most commonly obtained, as it prevents the formation of large aggregates and allows the self-assembly of asymmetric block copolymers with longer hydrophobic blocks. However, this method does not always prevent the formation of “frozen” micelles, due to the glass transition of the core at a certain temperature and/or in the presence of a given solvent [32]. Other conventional methods used for the preparation of drug-loaded micelles include the solution-casting method, emulsification, and the solvent evaporation method, as well as freeze-drying (lyophilization). In the solution-casting method, also known as the thin-film hydration method [40], the polymer and drug are dissolved in a volatile organic solvent, which is then evaporated, yielding the formation of a thin polymeric film, where polymer–drug interactions are favored. The subsequent hydration of the film with an aqueous solution led to the formation of micelles. The emulsification and solvent evaporation method [41] consists of physical entrapment of a hydrophobic drug using an oil-in-water (O/W) emulsion process, which involves dissolving the copolymer and drug in a non-water-miscible organic solvent (dichloromethane, ethyl acetate) followed by emulsification into an aqueous phase through sonication or high-shear homogenization. Solvent evaporation under reduced pressure then induces micelle formation through polymer precipitation. Lyophilization is often used as a post-processing step for stabilizing micelles and, in certain cases, as a direct preparation method using aqueous co-solvent systems (for example, tert-butanol/water). This technique enables prolonged storage of micelles and helps prevent drug degradation during storage. When rehydrated, micelles can reform if their structure is maintained during the drying process [38,42]. In recent years, advanced techniques for preparing polymer micelles for drug delivery have been developed. For example, microfluidic mixing enables the highly controlled assembly of nanocarriers by manipulating laminar flow and solvent mixing kinetics within microscale channels [43]. Additionally, supercritical fluid (SCF) processing has emerged as a promising approach. SCF utilizes the unique physicochemical properties of supercritical fluids, particularly carbon dioxide (scCO2) and trifluoromethane. These fluids exhibit liquid-like solvation and gas-like diffusivity above their critical temperature and pressure. These advancements contribute to the development of micellar systems that are more suitable for clinical application and pharmaceutical manufacturing [43].

### 2.3. Methods for Characterizing Polymer Micelles

**Figure 4:**
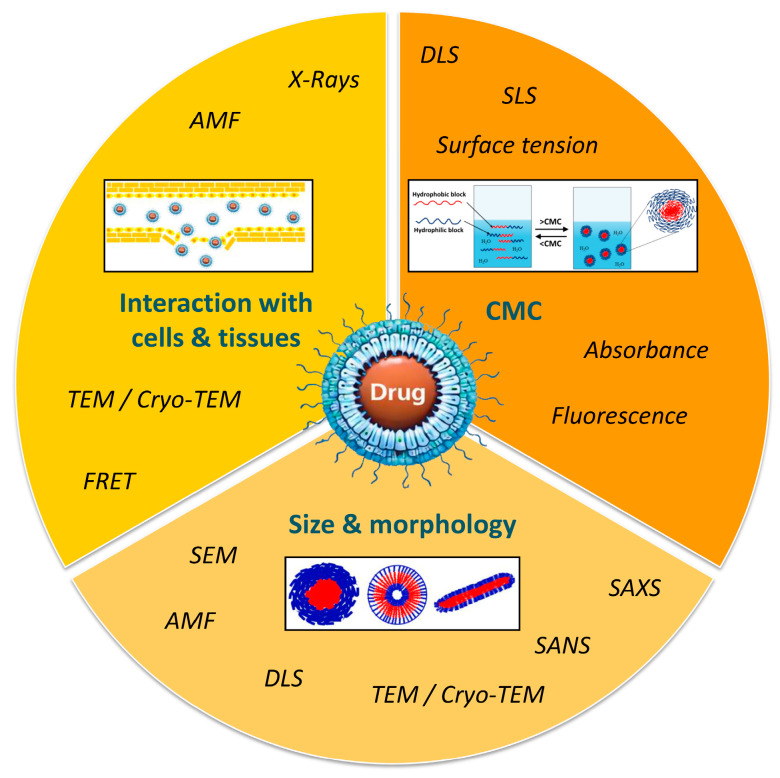
Methods for characterization of polymer micelles.

Micelle characterization is of critical importance and often requires the combination of different approaches and techniques in order to define and predict their behavior in a biological environment (Figure 4).

#### 2.3.1. Critical Micelle Concentration Determination (CMC)

CMC determination is an important aspect of polymer micelle characterization and represents the hydrophobic and hydrophilic balance in block copolymers. A low CMC indicates enhanced micelle stability, making them ideal for encapsulating hydrophobic drugs. Various methods are used to measure CMC, including light scattering, surface tension, and electrical conductivity based on macroscopic parameters, as well as photometric and fluorometric techniques that utilize suitable optical probes [44]. The determination of CMC by surface tension can be performed by the Wilhelmy plate method [45]. When the concentration of amphiphiles increases, the surface tension decreases until the CMC comes to a constant value. Surface tension is almost constant at a concentration of the polymer above the CMC value. However, this technique requires more time and a large number of samples [46]. Some of the most widely used methods are those based on light scattering. In the dynamic or quasi-elastic light scattering (DLS) method, the scattered light is influenced by the size and molecular mass of particles in micellar solutions. The intensity of light scattering remains relatively constant below the CMC, but increases significantly when the concentration reaches the CMC. This method also enables the determination of the hydrodynamic radius (RH) of polymer micelles based on their diffusion coefficient [47]. The fluorescence or absorbance technique is one of the most commonly used methods for determining the CMC by measuring the signal exhibited by dyes, such as pyrene or 1,6-diphenyl-1,3,5-hexatriene, within micelles formed by amphiphilic polymers. At certain polymer concentrations, a shift in excitation wavelength occurs due to the entrapment of dyes within the hydrophobic cores of polymer micelles [48,49].

#### 2.3.2. Morphological and Structural Characterization

The morphology and size of polymer micelles can be determined using transmission electron microscopy (TEM) and cryo-transmission electron microscopy (cryo-TEM). The primary difference between the two methods lies in their sample preparation for imaging. TEM requires complete dehydration of samples to ensure a high vacuum within the microscope column. In contrast, cryo-TEM involves rapidly freezing the samples at temperatures of at least −170 °C using liquid nitrogen, liquid propane, or liquid helium. This technique allows for the observation of specimens in their native state, preserving their solution structure and reducing artifacts that can occur during the removal of water [50,51,52,53]. Although cryo-TEM is recognized as a more suitable tool for imaging micelles compared to standard TEM, it is also time-consuming and expensive. As a whole, most TEM imaging of micelles relies on traditional TEM, as the differences in observations between the two methods are often not significant [54,55]. Another advanced technique for investigating the morphology of micelles is atomic force microscopy (AFM). This technique involves depositing the sample as a thin layer on a support and scanning it with a very sharp probe tip mounted on a cantilever. The tip deflects in response to the sample’s topography [56]. The tapping mode of AFM is especially favored for micelles, as it minimizes distortion of the micellar structure during analysis [57]. Small-angle X-ray scattering (SAXS) and small-angle neutron scattering (SANS) methods are two powerful tools used for the structural analysis of micelles. They allow determination of the micelle core size and hydrodynamic shell of polymer micelles in solution, and SANS also provides information for their cross-section [39,58]. To examine the interaction between micelles and biological environments, which is crucial for understanding their therapeutic efficacy, fluorescence-based techniques and Förster Resonance Energy Transfer (FRET) are commonly used. These methods allow for the investigation of how both physiological and pathological conditions affect micelle integrity, target site accumulation, biodistribution, and drug release kinetics [59].

## 3. Types of Polymer Micelles

Polymer micelles used in drug delivery technology can be categorized based on their responsiveness to environmental conditions. There are two main types: conventional or “non-responsive” micelles, which provide stable and long-term drug delivery mainly through passive diffusion, and “smart” or stimuli-responsive micelles, which enable targeted and controlled drug release in response to specific environmental triggers. These triggers can include physical, chemical, or biological stimuli.

### 3.1. Conventional (Non-Responsive) PEG-Based Polymer Micelles for Drug Delivery

These micelles are formed from amphiphilic block copolymers, typically diblock copolymers that do not have specific sensitivity to external stimuli. The most used hydrophilic block for the preparation of amphiphilic diblock copolymers for drug delivery is polyethylene glycol (PEG). PEG is favored due to its biocompatibility, excellent anti-fouling properties, and “stealth” functionality that helps minimize detection by the immune system, reducing immunogenicity [60]. The most commonly used polymers, as the hydrophobic blocks of the micelles, are typically polylactide (PLA), polycaprolactone (PCL), poly(lactic-co-glycolic acid) (PLGA), poly (amino acids), or lipids. These hydrophobic polymers form the inner core of the micelle and can serve as a depot for small drugs with poor water solubility [61]. The PEG-b-PLA, PEG-b-PCL, and PEG-b-PLGA block copolymers, which demonstrated excellent bio-degradability and biocompatibility, can be conveniently synthesized through the ring-opening polymerization of lactide, glycolide, or ε-caprolactone monomers, utilizing PEG as an initiator along with an appropriate catalyst [60,61]. Thus, the hydrophilic PEG shell of the micelles enhances micelle circulation time and reduces immune recognition, while the hydrophobic core effectively encapsulates hydrophobic drugs.

#### 3.1.1. Passive Targeting of Conventional Drug-Loaded Polymer Micelles

The passive targeting of drug-loaded micelles is a remarkable advancement in cancer therapy, driven by specific characteristics of tumor blood vessels, like their inherent leaky nature and poor lymphatic drainage. Among the various methods explored, the encapsulation of drugs within polymer micelles stands out as one of the most researched strategies for effective passive drug targeting. This approach exploits the enhanced permeability and retention (EPR) effect, allowing these nanosized micelles, which are less than 100 nm in diameter, to accumulate effectively in solid tumors. The disorganized structure of tumor blood vessels facilitates this accumulation, while the long circulation half-life of micelles ensures sustained presence at the site of action [62]. Extensive research has demonstrated that PEG-containing amphiphilic block copolymers significantly enhance the stability of these nanoparticles in systemic circulation and promote their selective accumulation in tumor tissues, all without the requirement of surface ligands or targeting moieties. Moreover, the ability of PEG to avoid recognition by the cells of the reticuloendothelial system (RES) allows for improved drug availability at the target site. Consequently, this mechanism not only maximizes the therapeutic potential of anticancer drugs but also enhances the probability of effectively reaching and treating the targeted tumors [63]. Various classes of anticancer chemotherapeutic drugs have been investigated as passive targeting polymer drug delivery systems, including taxanes, anthracyclines, platinum drugs, and natural compounds.

Taxane-Class Drugs’ Micellar Formulations

Taxanes are among the most widely used drugs in chemotherapy, for the treatment of various types of cancer, including breast, lung, esophageal, prostate, bladder, head, and neck cancers [64]. They inhibit mitosis, disrupt microtubule function, and obstruct depolymerization, which stops the cell division process [64]. Paclitaxel (Taxol^®^) and Docetaxel (Taxotere^®^) are among the most effective chemotherapeutic agents for the treatment of a wide range of solid tumors. However, their clinical application is limited by extremely poor aqueous solubility, the requirement for toxic solubilizing agents (e.g., Cremophor EL or polysorbate 80), dose-limiting hypersensitivity reactions, and severe systemic toxicities such as neurotoxicity [65]. To address these challenges, polymeric micellar formulations have emerged as an effective strategy by solubilizing taxanes within a hydrophobic core while forming a hydrophilic corona that prolongs systemic circulation and promotes tumor accumulation through the (EPR) effect. Several taxane-loaded micellar systems have advanced to clinical evaluation. The most prominent example is Genexol-PM^®^ (developed by Samyang Co., Seoul, Republic of Korea), which represents a lyophilized micellar formulation based on biodegradable poly(ethylene glycol)-block-poly(D, L-lactide) (PEG-b-PDLL) block copolymers and paclitaxel (PTX). This nanosized formulation (with a diameter of 20–50 nm) shows great promise in terms of water solubility and in vivo stability. The recommended phase II dosage for Genexol-PM^®^ was determined to be 300 mg/m^2^, as compared to 170 mg/m^2^ for Taxol^®^, thus achieving a higher paclitaxel dose without additional toxicity [66,67,68]. Docetaxel (DTX) has also been successfully encapsulated in methoxy-poly(ethylene glycol)-block-poly(D,L-lactide) polymer micelles, known as Nanoxel-PM™, which range in size from 20 to 50 nm. A preclinical study showed that Nanoxel-PM™ had fewer side effects, including hypersensitivity reactions and fluid retention, while demonstrating pharmacokinetic profiles and antitumor activity comparable to those of Taxotere^®^ [69]. Amphiphilic block copolymers composed of PEG and modified poly(aspartate), in which approximately half of the carboxyl groups were esterified with 4-phenyl-1-butanol to increase their hydrophobicity, were developed for efficient PTX encapsulation. Through self-assembly, PTX was physically entrapped within the hydrophobic micellar core via strong hydrophobic interactions, while the PEG corona imparted stealth properties. This PTX micellar formulation, known as NK105 (NanoCarrier™), is nonimmunogenic and suitable for intravenous administration without Cremophor EL or ethanol. In HT-29 human colorectal cancer xenograft models, NK105 exhibited significantly enhanced antitumor efficacy compared with free PTX, attributed to improved tumor accumulation and sustained drug release from the micelles. Moreover, NK105 demonstrated markedly reduced neurotoxicity relative to conventional paclitaxel formulations [70,71,72].

In addition, polyethylene glycol-b-poly(ε-caprolactone) (PEG-b-PCL) block copolymers (FDA-approved) have been extensively explored as polymeric nanocarriers for passive drug delivery. These amphiphilic copolymers exhibit high biocompatibility, controlled biodegradability, and self-assembly into micelles in aqueous media [12]. The low glass transition temperature of PCL further facilitates micelle formation and stable encapsulation of hydrophobic drugs [73]. The hydrophobic PCL core enables high loading of poorly soluble anticancer agents such as PTX and DTX, while the PEG corona provides steric stabilization and prolonged circulation [74]. A number of studies demonstrated the successful incorporation of paclitaxel or docetaxel into PEG-b-PCL micelles and their application in cancer therapy against breast [75], ovarian [76], and prostate [77] cancers, all of them demonstrating improved solubility, sustained release, enhanced antitumor efficacy, and reduced systemic toxicity in preclinical models compared to the free drug formulations.

*Anthracycline-Class Drugs’ Micellar Formulations*

Anthracyclines, a family of antitumor antibiotics, are another widely used class of chemotherapeutic agents, which are the most active cytotoxic agents for the treatment of a wide variety of solid tumors and hematological malignancies [78]. Despite their proven efficacy, their use is limited due to the toxicity to normal tissues and treatment resistance. The major side effect of anthracyclines is cardiotoxicity due to cumulative doses [79]. Among them, Doxorubicin (Adriamycin) plays an important role as a powerful anthracycline chemotherapeutic agent, which is widely used in oncology due to its ability to intercalate DNA and inhibit topoisomerase II, leading to the disruption of DNA replication and induction of apoptosis in rapidly dividing cells [80,81]. Clinically, doxorubicin is employed against a broad spectrum of hematopoietic malignancies (lymphoma, leukemia) and solid tumors such as breast and ovarian cancers [82]. Despite its broad antineoplastic activity, the clinical use of doxorubicin (DOX) is often limited by poor aqueous solubility, rapid systemic clearance, and dose-dependent cardiotoxicity. To overcome these limitations, polymeric micelles based on amphiphilic PEG-containing block copolymers have been extensively investigated as passive nanocarriers for doxorubicin delivery. For example, PEG-b-poly(ε-caprolactone) (PEG-PCL) micelles with encapsulated DOX (size of 36 nm) were prepared successfully by the solvent evaporation method. Thus, prepared micelles were tested against both drug-sensitive and multidrug-resistant models, including adriamycin-resistant K562 leukemia cells. After the initial burst release of the drug, a phase of significantly sustained drug release over a long period was observed. In comparison, doxorubicin solution was released completely only within 2 h. The results showed increased intracellular drug accumulation and enhanced cytotoxicity in both drug-sensitive and multidrug-resistant models, including adriamycin-resistant K562 leukemia cells, compared to the doxorubicin solution [83]. This indicates their ability to reverse multidrug resistance. Similarly, PEG-PCL-PEG triblock micelles have shown prolonged circulation and effective tumor suppression against drug-sensitive (MCF-7) breast cancer cell lines [84]. In addition, core cross-linked PCL-PEG-PCL micelles loaded with DOX have been tested on colon carcinoma cell models (C26 cell line in vitro), and results demonstrated that the encapsulated DOX in the micelles enhanced the cytotoxicity of DOX on the C26 cell line. Moreover, in vitro release profiles demonstrated a significant difference between the rapid release of free DOX and the much slower and sustained release of DOX-loaded core cross-linked micelles [85].

*Platinum-Class Drugs’ Micellar Formulations*

Platinum-based chemotherapeutics such as cisplatin and oxaliplatin are also widely used in oncology to treat various types of solid tumors, including ovarian, testicular, bladder, lung, and colorectal cancers. The mechanism of action involves covalent binding to purine DNA bases and the formation of cross-links that prevent DNA replication and transcription, thus leading to cellular apoptosis [86]. However, their clinical utility is limited by severe side effects (e.g., nephrotoxicity, neurotoxicity) and the development of resistance. Cisplatin is highly active and widely used for the treatment of a variety of cancers; however, its use in practice is limited due to cumulative dose-limiting toxicities (DLTs) and a lack of improvement in efficacy despite longer treatment [87]. While highly effective, the use of cisplatin is associated with irreversible ototoxicity and nephrotoxicity [88]. To improve their therapeutic index and exploit passive targeting via the EPR effect, platinum drugs have been incorporated into PEG-based polymeric micelles that prolong circulation and facilitate tumor accumulation. Kataoka and coworkers designed a series of PEG-b-poly(amino acid)-based micelles loaded with cisplatin for passive drug targeting into tumors. The carboxylic groups in the poly(amino acid) can form a complex with cisplatin, oxaliplatin, or other organometallic compounds via coordination [89,90]. The optimization of cisplatin-loaded PEG-poly(glutamic acid) micelles results in the development of Nanoplatin™ (NC-6004), a polymeric micelle where cis-diamminedichloroplatinum(II) is complexed with the carboxylate groups of PEG-poly(glutamic acid), leading to enhanced stability in aqueous solutions and improved drug release characteristics. The free drug is regenerated in the presence of chloride ions. Compared with free cisplatin, NC 6004 delivers higher amounts of cisplatin to solid tumors, while also being taken up by the liver and spleen. Preclinical studies demonstrated comparable antitumor activity with reduced nephrotoxicity. Clinically, NC 6004 exhibits prolonged circulation, enhanced tumor accumulation, and disease stabilization in patients with solid tumors, including advanced colorectal cancer, while minimizing typical cisplatin-associated toxicities [91,92,93].

In addition, the active oxaliplatin analog, (1,2-diaminocyclohexane)platinum(II) (DACHPt), has been incorporated into PEG-b-PGlu block copolymer micelles, which vary in PGlu length. In vivo distribution and antitumor activity experiments conducted on CDF1 mice bearing murine colon adenocarcinoma (C-26) showed that these micelles accumulate at the tumor site at levels 20 times greater than oxaliplatin, achieving significantly higher antitumor efficacy. The strong antitumor activity of DACHPt-loaded micelles was demonstrated, exhibiting very effective results against multiple metastases generated from injected bioluminescent HeLa (HeLa-Luc) cells [94,95]. The similar DACHPt-micelles were demonstrated to have efficient penetration and accumulation in an orthotopic scirrhous gastric cancer model, leading to the inhibition of the tumor growth. Moreover, the elevated localization of systemically injected DACHPt-micelles in metastatic lymph nodes can inhibit the growth of metastatic tumors [94,95,96].

Recent studies have investigated PEG-b-PCL and related micellar systems for the delivery of cisplatin, showing enhanced antitumor efficacy both in vitro and in vivo. These systems demonstrated improved biodistribution and reduced systemic toxicity compared to free cisplatin [97]. The biodistribution, antitumor efficacy, and toxicity of cisplatin-loaded core cross-linked micelles based on poly(ethylene glycol)-b-poly(methacrylic acid) (PEG-b-PMA) were also evaluated using a mouse model of ovarian cancer. The cisplatin-loaded micelles showed prolonged blood circulation, increased accumulation in tumors, and reduced exposure to the kidneys. Compared to the free drug, the micelles exhibited an improved antitumor response in this mouse model [98].

*Natural compound Drugs’ Micellar Formulations*

Camptothecin (CPT), a natural alkaloid with significant antiproliferative activity, and its derivatives, such as SN 38 and irinotecan, are potent topoisomerase I inhibitors—an essential enzyme for DNA replication, transcription, and repair processes [99] with broad anticancer activity, including colorectal, lung, breast, and other solid tumors [100]. The clinical application of camptothecin has been limited due to its systemic toxicity, poor aqueous solubility, and the instability of the lactone ring under physiological conditions. These factors significantly reduce its efficacy. To improve pharmacokinetics and take advantage of passive targeting through the EPR effect, various PEG-based polymer micelles have been developed to encapsulate camptothecin compounds. For example, one study involved the physical entrapment of camptothecin in a block copolymer based on mPEG-p(β-benzyl L-aspartate) (PEG-PBLA). The formulation’s efficacy and size were optimized by adjusting the drug-to-polymer ratio. Additionally, the stability of the formulation in vivo was affected by the length of the mPEG block and the amount of benzyl ester, resulting in an extended circulation time [101]. Such PEG poly(benzyl aspartate) micelles have successfully delivered parent CPT in murine colon cancer (C26) xenografts, increasing plasma and tumor drug levels by approximately 150 and five-fold, respectively, compared with free CPT, leading to significantly higher tumor growth inhibition [102]. Further development has focused on creating ideal nanoparticles that protect camptothecin and prevent the hydrolysis of its lactone form. A copolymer of PEG-PCL was chosen to prepare polymeric micelles through a solvent evaporation method, resulting in optimal size and effective encapsulation of camptothecin. The nanoparticles were subsequently coated with a red blood cell (RBC) membrane, which provides “stealth” properties, enhancing circulation time and protecting camptothecin from the reticuloendothelial system (RES) and hydrolysis. When evaluating the hydrolysis rate of the nanomicelles—both with and without the RBC coating—results showed a slower rate of hydrolysis. After 1 h, only 25% of the drug had undergone hydrolysis, while after 6 h, 64% of the active lactone form of camptothecin remained intact [103]. In addition to parent CPT, the highly potent derivative SN 38 has been formulated in mixed PEG-based micellar systems by combining PEG b PCL with other amphiphiles such as Pluronic F-108 to increase the solubility of SN-38. A clear and stable micellar system with enhanced drug loading was prepared, and high antitumor efficacy against MCF-7 breast cells was demonstrated [104].

Curcumin (CUR) is a natural hydrophobic polyphenol with demonstrated anticancer activity against various solid tumors, including colon carcinoma, breast carcinoma, and non-small cell lung cancer (NSCLC). Its clinical application is limited by poor aqueous solubility and rapid metabolism. To address these issues, methoxy poly(ethylene glycol)-poly(ε-caprolactone) (mPEG-b-PCL) micelles have been used to encapsulate curcumin in the hydrophobic core, improving solubility, pharmacokinetics, and sustained release. These micelles significantly inhibited CT26 colon carcinoma growth in vitro and in vivo and enhanced anti-angiogenic effects compared with free CUR [105]. Similarly, mPEG-b-PLA micelles loaded with curcumin increased cellular uptake and cytotoxicity in A549 NSCLC cells as well as murine melanoma (B16F10) and human breast cancer (MDA MB 231) cells, induced apoptosis, and suppressed migration and invasion more effectively than free CUR [106,107]. Beyond single-agent systems, co-delivery of curcumin with other drugs such as doxorubicin in PEG-based micelles has been shown to reverse multidrug resistance and enhance antitumor efficacy in models of drug-resistant breast cancer, although those systems combine active agents rather than solely demonstrating passive delivery of CUR [108].

#### 3.1.2. Active Targeting of Conventional Drug-Loaded PEG-Based Polymer Micelles

Passive targeting strategies have shown several limitations, including heterogeneous tumor vasculature, variable EPR effect among tumor types, and nonspecific distribution in healthy tissues. Therefore, considerable effort is being directed toward maximizing nanoparticle accumulation and therapeutic efficacy through active targeting strategies. Site-specific delivery and cellular internalization can be achieved by functionalizing nanoparticles with ligands that recognize overexpressed receptors on tumor cell membranes, or by exploiting phagocytosis and receptor-mediated endocytosis mechanisms, thereby improving selective uptake and minimizing off-target effects [109]. PEG-based polymer micelles are widely employed for targeted drug delivery due to their excellent biocompatibility, prolonged circulation time, and ease of surface functionalization. The hydrophilic PEG corona provides steric stabilization and reduces nonspecific protein adsorption, while terminal functional groups on PEG chains enable conjugation of targeting ligands. Commonly used ligands include folic acid, RGD peptides, transferrin, antibodies or antibody fragments, and aptamers, which recognize receptors overexpressed on cancer cells or tumor vasculature [11,13]. Ligand-functionalized PEG micelles promote receptor-mediated endocytosis, leading to enhanced cellular uptake, increased tumor accumulation, and improved therapeutic efficacy while minimizing systemic toxicity compared to non-targeted micelles.

*Folic acid (FA)* is widely used due to its favorable properties, including its small size, nonimmunogenicity, low toxicity, and ease of conjugation. FA-functionalized micelles can efficiently deliver various anticancer agents into target tumor cells, thereby reducing nonspecific interactions with healthy tissues and enhancing site-specific drug action [110,111]. The folic receptors (FR) have been known to overexpress in several human tumors, including ovarian and breast cancers, while they are highly restricted in normal tissues [4]. Several studies have demonstrated that folate-conjugated polymer micelles exhibit enhanced cytotoxicity and cellular uptake in folate receptor FR-positive cancer cells compared to non-folate-functionalized micelles. For instance, Park et al. developed folate-conjugated MPEG-b-PCL micelles loaded with paclitaxel (PTX), with particle sizes ranging from 50 to 130 nm depending on the molecular weight of the block copolymers. The in vitro release profile of paclitaxel from these micelles showed sustained drug release without an initial burst, indicating controlled delivery. Furthermore, paclitaxel-loaded folate-conjugated MPEG-b-PCL micelles exhibited significantly higher cytotoxicity against FR-positive cancer cell lines, including MCF-7 and HeLa cells, compared to micelles lacking folate, highlighting the effectiveness of ligand-mediated targeting in enhancing therapeutic outcomes [112]. Folate-decorated diblock copolymers based on poly(D,L-lactic-co-glycolic acid) (PLGA) and poly(ethylene glycol) (PEG) loaded with doxorubicin (DOX) and free DOX mixed micelles have also been developed for targeted drug delivery. In vivo studies using a nude mouse xenograft model implanted with a human epidermal carcinoma xenograft cell line, KB cells, demonstrated that the systemic administration of these micelles led to significant tumor regression against KB cells (folate receptor positive), indicating effective tumor-targeted delivery and enhanced therapeutic efficacy [113]. Using a similar PLGA-PEG-FOL system, Zhao et al. further evaluated the selectivity and cytotoxicity of DOX-loaded micelles across cancer cell lines with varying folate receptor levels (KB, MATB III, and C6) and normal fibroblasts (CCL-110). The study showed that cytotoxicity was substantially higher in cancer cells than in normal fibroblasts, and cell cycle analysis confirmed a lower percentage of apoptotic normal cells, demonstrating the ability of folate-conjugated micelles to selectively target tumor cells while minimizing toxicity to healthy tissues [114]. Folic acid-modified PEG-PLGA (FA-PEG-PLGA)-based micelles were also developed for the co-delivery of cisplatin (CDDP) and paclitaxel (PTX), which were encapsulated in the hydrophobic core and chelated to the middle shell, respectively, while PEG formed the outer corona for prolonged circulation. In vitro, the dual-drug-loaded nanomicelles showed a highly synergistic inhibition of both FA receptor-negative A549 and FA receptor-positive M109 lung cancer cells. In vivo, CDDP+PTX nanoparticles achieved tumor suppression rates of 89.96% for A549 xenografts and 95.03% for M109 xenografts, which are significantly higher than those of free chemotherapy drug combinations or nanoparticles with a single drug. These results indicate that FA-PEG-PLGA-based co-delivery of CDDP and PTX offers an effective and safe strategy for targeted cancer chemotherapy, particularly for FA receptor-expressing tumors [115]. In addition, folate-targeted mixed micelles were also prepared by folate-poly(ethylene glycol)-distearoylphosphatidylethanolamine (FA-PEG-DSPE) and methoxy-poly(ethylene glycol)-distearoylphosphatidylethanolamine (MPEG-DSPE) to encapsulate the anticancer agent 9-nitro-camptothecin (9-NC). These micelles were evaluated against three tumor cell lines (HeLa, SGC7901, and BXPC3). The optimal molar ratio of FA-PEG-DSPE to MPEG-DSPE was determined to be 1:100, which allowed for efficient solubilization of 9-NC, reduced uptake by macrophages in vitro, and exhibited higher antitumor activity to the tumor cells (pancreatic cancer cell line, human uterine cervix cancer cell line, and human gastric cancer cell line) with overexpressed folate receptors on cell surface in comparison with folate-free micelles or free anticancer agents [116].

*RGD-containing peptides* are also widely used as tumor-targeting ligands, which were first developed by Kessler et al. [117] to target the αvβ_3_ and αvβ_5_ integrin receptors [118]. Integrins are heterodimeric transmembrane receptors composed of alpha (α) and beta (β) subunits that mediate cell–extracellular matrix (ECM) and cell–cell adhesion. They serve as physical and functional links between the ECM and cytoskeletal control pathways, transducing bidirectional signals across the plasma membrane [119]. These receptors are overexpressed in angiogenic tumor endothelial cells, as well as in a wide variety of solid tumors [120]. Poly(ε-caprolactone)-b-poly(ethylene glycol) (PCL-b-PEG) block copolymer micelles loaded with doxorubicin (DOX) and surface-conjugated with the cyclic pen-tapeptide c (Arg-Gly-Asp-D-Phe-Lys) (cRGD) were developed for the selective delivery of DOX to angiogenic tumor endothelial cells overexpressing αvβ3 integrins. This targeting strategy exploits the high binding affinity and selectivity of cRGD toward the αvβ_3_ integrin. Remarkable increase in the uptake of micelles in the SLK cells upon attachment of cRGD molecules to the micelle surface was observed, and maximum ~30-fold enhancement in cellular uptake was achieved with 76% cRGD-functionalized DOX-loaded micelles compared with non-functionalized DOX-loaded micelles, demonstrating the strong potential of cRGD-mediated targeting for angiogenesis-directed cancer therapy [121]. Zhan et al. evaluated cRGD-conjugated PEG-b-PLA micelles as drug carriers for paclitaxel to treat integrin αvβ_3_ overexpressed glioblastoma. It was shown that while the drug-loaded PEG-PLA micelles and Taxol^®^ arrested tumor growth in mice bearing U87MG s.c. xenografts, cRGD-conjugated paclitaxel micelles exhibited the most potent tumor growth inhibition [122]. The treatment with cRGD-conjugated paclitaxel micelles resulted in the longest survival time in intracranial U87MG tumor-bearing mice. Similarly, RGD-functionalized PEG-b-PLA micelles loaded with curcumin (Cur-RPP) were prepared using the thin-film hydration method. The cellular uptake of Cur-RPP was significantly higher than that of non-RGD-modified micelles, due to the specific binding between the αvβ3 integrin and RGD ligands in human umbilical vein endothelial cells (HUVECs) and mouse melanoma B16 cell lines. In B16 tumor-bearing mice, Cur-RPP exhibited a stronger tumor growth inhibition compared with non-RGD-modified micelles, highlighting the effectiveness of RGD-mediated targeting in enhancing therapeutic efficacy [123].

*Transferrin (Tf)* is another ligand used for active drug delivery systems. It is a cell membrane-associated glycoprotein involved in the cellular uptake of iron and in the regulation of cell growth. Iron uptake occurs via internalization of iron-loaded transferrin mediated by the transferrin receptor (TfR). As a targeting moiety, transferrin triggers receptor-mediated endocytosis in cells that highly express TfR, which include many cancer types [124]. For example, transferrin (Tf) was conjugated onto the surface of appropriately modified PEG-b-PLA block copolymer micelles with loaded paclitaxel (PTX) to enable active tumor targeting. The antitumor efficacy of these Tf-functionalized micelles was evaluated in gastric carcinoma models overexpressing the transferrin receptor (TfR). Analysis of tumor volume progression, together with histological examination of hematoxylin and eosin-stained tumor sections, demonstrated that Tf-modified PTX-loaded micelles exhibited the strongest antitumor activity compared with the control groups, including saline, free PTX, and non-targeted PTX-loaded micelles [125]. More recently, a glioma-targeted drug delivery system was developed using PTX-loaded PEG-PLA polymeric micelles decorated with the transferrin receptor-targeting peptide TfR-T12, enabling efficient transport across the blood–brain barrier (BBB) and specific recognition of glioma cells. In vitro cellular uptake and cytotoxicity studies demonstrated significantly enhanced internalization of TfR-T12/PEG-PLA micelles in U87MG glioma cells, accompanied by superior anticancer activity compared with non-targeted micelles. This enhanced performance was attributed to the presence of the TfR-T12 ligand on the micelle surface, which selectively binds to the overexpressed transferrin receptors (TfRs) on U87MG cells. In vivo biodistribution and therapeutic efficacy studies in tumor-bearing mice further confirmed the advantages of this targeting strategy. Following intravenous administration, TfR-T12/PEG-PLA-PTX micelles exhibited improved tumor accumulation and significantly enhanced antitumor activity, as evidenced by reduced tumor cell proliferation and angiogenesis, along with increased apoptosis, compared with non-targeted PEG-PLA-PTX micelles [126].

*Antibodies* offer the greatest versatility due to their broad target range and high binding specificity. Antibody-targeted micelles, or immunomicelles, can be prepared by chemically attaching antibodies or antibody fragments to the activated, water-exposed ends of the hydrophilic block of the micelle-forming polymer. This functionalization enables the micelles to selectively bind antigens or receptors overexpressed on tumor cells, enhancing targeted drug delivery and therapeutic efficacy [5,127]. It was shown that certain non-pathogenic monoclonal antinuclear autoantibodies with nucleosome-restricted specificity, such as monoclonal antibody 2C5 (mAb 2C5), selectively recognize the surface of a wide range of tumor cells, but not normal cells, via tumor cell surface-bound nucleosomes. Due to their ability to bind diverse cancer cells, these antibodies can serve as specific ligands for the targeted delivery of drugs and nanocarriers into tumors, enhancing therapeutic efficacy while minimizing off-target effects. By adapting the coupling technique, PEG-phosphatidylethanolamine (PE)-based immunomicelles modified with mAb 2C5 with nucleosome-restricted specificity reactive toward a variety of different cancer cells were developed [128]. The intravenous administration of tumor-specific 2C5 immunomicelles based on PTX-loaded PEG-PE into experimental mice bearing murine Lewis lung carcinoma resulted in an increased accumulation of PTX in the tumor compared with free drugs or drugs in non-targeted micelles and in an enhanced tumor growth inhibition in vivo [129]. In another more recent example, doxorubicin (DOX)-loaded polymeric immunomicelles made from poly(d,l-lactic-co-glycolic acid)-PEG (DOX–PLGA-PEG) and targeted with the bivalent fragment HAb18 F(ab′)2 against hepatocellular carcinoma (HCC) were developed. The therapeutic action of micelles improved owing to specific molecular targeting and a higher uptake by the HCC cell lines HepG2 and Huh7 in vitro. The targeted micelles also suppressed in vivo tumor growth significantly (63.9%) when compared to free DOX (39.8%) or DOX-PLGA-PEG (50.2%) in HepG2 xenograft-bearing nude mice [130].

*Epidermal Growth Factor Receptor (EGFR)* is a cell-surface receptor for the members of the epidermal growth factor family (EGF-family) and is overexpressed on the surface of a number of different human cancer cells, including colorectal, breast, and lung cancer cells [131]. Recently, the possibility of using an EGF-conjugated polymer micelle as a vehicle for targeting hydrophobic drugs to EGFR-overexpressing cancers has been investigated. For example, Zeng et al. [132] reported an EGF-conjugated PEG-b-poly (d-valerolactone) (PGG-b-PVL) micelle system that targets the EGF receptors overexpressed by breast cancer cells. These micelles were shown to localize in the nucleus of the MDA-MB-468 breast cancer cells and the perinuclear region. Thus, EGF conjugates are useful for nuclear targeting, which is critical for the delivery of anticancer drugs whose site of action is located in the nucleus. In another system, anti-EGFR antibodies were conjugated to PEG-b-PCL block copolymer micelles for active targeting of EGFR-overexpressing cancer cells. Doxorubicin (DOX) was encapsulated in the micelle core (DOX-micelle), and RKO colorectal cancer cells were treated with free DOX, DOX-micelles, or DOX-anti-EGFR-micelles. Free DOX is primarily localized to the nuclei, whereas DOX-micelles accumulate in the cytoplasm. Notably, DOX-anti-EGFR-micelles induced significantly higher apoptosis than free DOX or non-targeted DOX-micelles, demonstrating that antibody-functionalized micelles are an effective strategy for targeted delivery of cytotoxic drugs to EGFR-overexpressing cancer cells [133].

*Aptamers (Apts)* offer several distinct advantages, including nonimmunogenicity, small molecular size, ease of chemical synthesis and modification, and high batch-to-batch reproducibility compared to antibodies. They are DNA or RNA oligonucleotides that fold into well-defined three-dimensional structures through intramolecular interactions, enabling them to bind target molecules with high affinity and specificity in a manner analogous to antibodies [134]. Since their discovery through in vitro selection techniques, aptamers have attracted significant attention as targeting ligands in drug delivery systems, since they exhibit remarkable physicochemical stability, retaining their binding activity over a broad range of pH values (≈4–9), temperatures, and even in the presence of organic solvents. These favorable properties make aptamers particularly attractive as targeting moieties for nanoparticle and polymer micelle-based drug delivery applications [135]. For example, docetaxel (Dtxl)-loaded PLGA-b-PEG polymeric micelles functionalized with A10 2′-fluoropyrimidine RNA aptamers targeting prostate-specific membrane antigen (PSMA) were developed for prostate cancer therapy. The aptamer-decorated micelles exhibited significantly enhanced in vitro cytotoxicity compared with non-targeted nanoparticles. Following a single intratumoral administration in LNCaP xenograft nude mice, the targeted formulation achieved complete tumor regression in five out of seven animals and 100% survival over 109 days, markedly outperforming non-targeted micelles and free Dtxl. In contrast, saline and drug-free nanoparticles showed no therapeutic efficacy [136]. Similarly, Pt(IV) compound (hydrophobic Pt(IV) prodrug with axial alkyl chains) was successfully loaded into PEG-b-PLGA micelles functionalized with the A10 2′-fluoropyrimidine RNA aptamer targeting PSMA. Following cellular uptake, intracellular reduction in the Pt(IV) prodrug resulted in the controlled release of active cisplatin. In vitro cytotoxicity assays performed in PSMA-positive LNCaP and PSMA-negative PC3 cells demonstrated that PSMA aptamer-targeted Pt(IV)-encapsulated PLGA-b-PEG micelles exhibited significantly enhanced anticancer activity. Notably, in PSMA-positive LNCaP cells, the targeted nanoparticles were approximately 80-fold more cytotoxic than free cisplatin, while showing significantly lower toxicity toward PSMA-negative PC3 cells [137].

### 3.2. Conventional PVP-Based Polymer Micelles for Drug Delivery

#### 3.2.1. Passive Targeting of PVP-Based Polymer Micelles for Drug Delivery

Although PEG-based micelles are among the most widely investigated drug delivery systems for cancer therapy, a recent study demonstrated that the multiple use of PEGylated drugs can lead to the development of anti-PEG antibodies (APAs), thereby accelerating drug clearance, decreasing therapeutic efficacy, and increasing the risk of adverse reactions, such as hypersensitivity. Pre-existing APAs have also been found in people without previous exposure to PEGylated drugs, which raises additional clinical concerns [138]. An alternative to PEG is poly(N-vinylpyrrolidone) (PVP), which can be effectively utilized as an appropriate hydrophilic polymer in drug delivery systems. PVP is known for its excellent biological and physicochemical properties, such as high water solubility, low toxicity, biocompatibility, complexation capability, cryo-protectivity, lypoprotectivity, and anti-biofouling properties [139]. However, very few reports on the synthesis and characterization of PVP-based copolymers as drug delivery micellar systems for cancer therapy are available in the literature. More research is needed to better understand their potential and to demonstrate their importance in modern nanomedicine.

*Taxane-Class Drugs’ Micellar Formulations*

Poly(N-vinylpyrrolidone)-block-poly(D,L-lactide) (PVP-b-PDLLA) copolymer was successfully used as a nano-micellar drug carrier for taxanes such as paclitaxel (PTX) and docetaxel (DCTX). PTX-loaded micelles were chosen as a model and evaluated in vitro on three different cancer cell lines: mammary carcinoma tumor EMT-6 cells, murine colon adenocarcinoma tumor C26, and human OVCAR-3 cells. The cytotoxicity experiments showed that each cell line exhibited different sensitivities to the drug, as the OVCAR-3 cells were the most sensitive to PTX, in contrast to C26 cells, which were relatively resistant to this drug. The antitumor activity of the PTX-loaded micelles against solid tumors tested in vivo on mice bearing murine C26 colon adenocarcinoma cells demonstrated that the maximum tolerated dose (MTD) was not reached, even at 100 mg/kg, in comparison to the MTD of Taxol^®^, where the established value was 20 mg/kg. At a 60 mg/kg dosage, PM-PTX demonstrated greater in vivo antitumor activity than Taxol^®^ injected at its MTD [140]. Next, triblock PVP-PDLL-PVP copolymers and star-(PDLLA-b-PVP)_4_ copolymers were evaluated as drug micellar carriers of PTX. It was determined that the self-assembling behavior and loading efficiency of PTX depend on their composition [141]. Similarly, paclitaxel (PTX) was loaded into poly(N-vinylpyrrolidone)-b-poly(ε-caprolactone) (PVP-b-PCL) micelles via a modified nano-precipitation method. The antitumor effect of PTX-loaded micelles was evaluated in vitro on three different cancer cell lines, including human gastric carcinoma cell line BGC 823, human oral epidermoid carcinoma cell line KB, and murine hepatic carcinoma cell line H22. The observed differences in the cytotoxicity among the tested cell lines are likely due to their genetic backgrounds and biological behaviors. The in vivo examinations tested on a hepatic H22 tumor-bearing mice model (i.v.) exhibit a significantly superior antitumor effect than the commercially available Taxol^®^ formulation [142].

*Anthracycline-Class Drugs’ Micellar Formulations*

A well-defined poly(N-vinylpyrrolidone)-b-poly(ε-caprolactone) (PVP-b-PCL) copolymer was synthesized by ROP of CL and controlled metal-free xanthate-mediated RAFT polymerization of NVPA. This copolymer was used to encapsulate doxorubicin (DOX) via the dialysis method, resulting in the formation of DOX-loaded micelles. The micelles showed enhanced growth inhibition and cytotoxicity against both parental and DOX-resistant human (K-562, JE6.1, Raji) as well as mice lymphoma cells (DL) (Dalton’s lymphoma, DL). They demonstrated a higher tumoricidal effect against DOX-resistant tumor cells compared to free DOX. It was found that additional treatment with DOX-loaded micelles did not affect the viability of normal blood cells, such as monocytes, dendritic cells, or lymphocytes (93.26%), whereas free DOX reduces their viability to 60.87% [139].

Additionally, mixed polymer micelles based on poly(vinyl pyrrolidone-b-polycaprolactone) (PVP-b-PCL) and poly(vinyl pyrrolidone-b-poly(dioxanone-co-methyl dioxanone)) (PVP-b-P(DX-co-MeDX)) copolymers were also prepared. These micelles were successfully loaded with various anticancer drugs from different classes, including gemcitabine (GEM) doxorubicine.HCl (DOX.HCl), doxorubicin. NH_2_ (DOX), 5-fluorouracil (5-FU), and paclitaxel (PTX). Hydrophobic drugs demonstrated a higher loading percentage efficiency compared to hydrophilic drugs, with the following trend: PTX > DOX > 5-FU > GEM > DOX.HCl. However, the drug release pattern followed the opposite trend due to a decrease in polymer–drug interaction. The physically mixed GEM and DOX.HCl-loaded micelles were selected as a model and tested against PANC-1 and BxPC-3 pancreatic cancer cell lines. The results demonstrated greater toxicity against PANC-1 and BxPC-3 cell lines compared to mixed free drugs and single-loaded micelles. This observation is probably due to their similar size and release kinetics profiles, which enhance the synergistic or additive drug effect of the drugs [143].

*Natural compound Drugs’ Micellar Formulations*

Tetrandrine (Tet) is a bis-benzylisoquinoline alkaloid isolated from the root of hang-fang-chi (Stephania tetrandra S Moore). It was identified as a promising anticancer drug due to its antitumor effectiveness against a wide range of cancers, including lung, breast, colon, liver, prostate, gastric, ovarian, pancreatic, cervical, bladder, and glioma cancers [144]. In this respect, poly(N-vinylpyrrolidone)-block-poly(ε-caprolactone) (PVP-b-PCL)-based micelles with high loading efficiency of Tet were prepared via the nanoprecipitation method. The cellular uptake test conducted on human non-small cell lung cancer cell line A549 showed that the uptake of Tet-NPs is mainly mediated by the endocytosis of the micelles and causes their apoptosis by inhibiting the expression of anti-apoptotic Bcl-2 and Bcl-xL proteins. These results confirm the potential of Tet-loaded micelles in lung cancer treatment and represent an effective strategy for improving its anticancer efficacy [145].

Recently, amphiphilic derivatives of poly-*N*-vinylpyrrolidone, with terminal hydrophobic long-chain n-alkyl fragments of different lengths, have gained attention due to their ability to self-assemble in aqueous media. These amphiphiles can form core–shell type polymeric nanoparticles, which are effective to entrap various hydrophobic biologically active agents in their inner core [146].

In this respect, poly-N-vinyl-2-pyrrolidone with thiooctadecyl end-group (PVP-OD) was synthesized and used as a nanocarrier for curcumin. Emulsification and ultrasonic dispersion methods were applied for the formation of curcumin-loaded nanocarriers and tested on two cell lines: U87 glioblastoma and CRL 2429 fibroblast cells as model systems. It was found that this delivery system exhibits two distinct mechanisms of cell penetration, depending on the preparation methods, by endocytosis mechanisms or by diffusion through the cell membrane via non-endocytic mechanisms. As a result, by precisely tailoring the size of polymeric carriers, they can effectively deliver hydrophobic drugs to all cell compartments, including the nucleus [147].

#### 3.2.2. Active Targeting of Drug-Loaded PVP-Based Micelles

Tumor necrosis factor (TNF)-related apoptosis-inducing ligand (TRAIL) is a type II transmembrane protein and a member of the TNF family. It induces apoptosis in transformed or tumor cells, making it a promising selective anticancer agent [148]. Therefore, amphiphilic poly(N-vinylpyrrolidone) nanoparticles consisting of unmodified and maleimide-modified polymeric chains (1:1) were covalently conjugated with antitumor DR5-specific TRAIL variant DR5-B to overcome the receptor-dependent TRAIL-resistance of tumor cells. The cytotoxicity of the nanoparticles was studied in 2D and 3D in vitro models, including human breast adenocarcinoma MCF-7 cells, human colorectal carcinoma HCT116, and colorectal adenocarcinoma HT29 cells. These formulations were found to enhance cytotoxicity effects compared to the free DR5-B in both 2D (monolayer culture) and 3D (tumor spheroids) in vitro models. Importantly, the conjugation of DR5-B with Amph-PVP nanoparticles increased the sensitivity of resistant multicellular tumor spheroids derived from MCF-7 and HT29 cells. However, further improvement is necessary to create a versatile system for targeted drug delivery using click chemistry [149]. More recently, amphiphilic PVP nanoparticles were loaded with bortezomib (BTZ)—a proteasome inhibitor—and further decorated with the TRAIL variant DR5-B (PVP-BTZ-DR5-B). The cytotoxicity of the nanoparticles was studied in vitro on 2D and 3D cultures of human glioblastoma cell lines U87MG and T98G. The results demonstrated that PVP-BTZ-DR5-B nanoparticles were internalized and accumulated in the cells more efficiently, demonstrating significantly enhanced cytotoxicity compared to free DR5-B or PVP-BTZ nanoparticles. Additionally, they penetrated the blood–brain barrier more effectively than DR5-B. The enhanced antitumor effect of PVP-BTZ-DR5-B was also demonstrated in a xenograft model of U87MG glioblastoma cells using zebrafish embryos in vivo. Therefore, the present system is a promising approach to enhance the antitumor efficacy of free drugs and overcome glioblastoma resistance [150]. In another example, amphiphilic N-vinylpyrrolidone nanoparticles with bortezomib (BTZ) were modified either with DR5-selective TRAIL cytokine (DR5-B) or its fusion with the iRGD peptide (DR5-B-iRGD), resulting in AmphPVP-BTZ-DR5-B and AmphPVP-BTZ-DR5-B-iRGD formulations. The cytotoxicity of the nanoparticles was tested on pancreatic adenocarcinoma cell lines PANC-1, BxPC-3, and MIA PaCa-2. Both types of nanoparticles were found to inhibit the growth of pancreatic adenocarcinoma cell lines to a great extent. Additionally, they promoted a more rapid internalization of the DR5 receptor in MIA PaCa-2 cells compared to unmodified particles and free forms of DR5-B or DR5-B-iRGD. Notably, AmphPVP-BTZ-DR5-B-iRGD demonstrated a more pronounced rate of DR5 internalization and a stronger cytotoxic effect than AmphPVP-BTZ-DR5-B, which can be attributed to the inclusion of a fusion protein containing the internalizing iRGD peptide. High cytotoxicity against pancreatic adenocarcinoma cells without significant cytotoxicity on healthy cells makes them promising candidates for pancreatic cancer therapy [151].

## 4. Stimuli-Responsive Polymer Micelles

Stimuli-responsive micelles are formed using intelligent polymers, commonly known as “smart” or stimuli-responsive polymers. What makes these polymers unique is their ability to undergo significant changes in characteristics with only slight variations in external factors, such as temperature, pH levels, or the presence of enzymes or biomolecules. The primary focus in this review is on pH-sensitive and temperature-sensitive PEG-based micelles derived from their respective stimuli-responsive polymers.

### 4.1. pH-Sensitive PEG-Based Polymer Micelles

The extracellular pH of normal tissues and blood is 7.4, which tends to be lower in tumor tissues. The pH of most extracellular tumors has values between pH 6.5 and 7.2. However, the pH can even be down at the intracellular level. The reported values range from 5.0 to 6.0 in endosomes and 4.0 to 5.0 in lysosomes. For this reason, the acidity in the extracellular and intracellular compartments constitutes an essential signal for targeting [152].

pH-sensitive polymer micelles are obtained from amphiphilic block copolymers whose polymer chains contain functional groups capable of accepting or donating protons upon changes in the pH of the environment. They are part of the so-called “smart” polymer systems, which can respond to changes in the external pH. Depending on the nature and location of the pH-responsive groups in the polymer, pH-sensitive polymers are classified into two main groups: (i) Polymers that contain ionizable groups in the side chain of the polymer, which are further divided into cationic and anionic polymers [153], and (ii) polymers that contain acid/base-labile linkages in the polymer chain. These are generally stable at pH 7.4 but hydrolyze under mild acidic conditions. The most commonly used acid-labile linkages include hydrazones, acetals/ketals, oximes, orthoesters, and cis-aconityl [154,155].

#### 4.1.1. Polymeric Nanomicelles for Tumor pH Targeting by a Destabilization Mechanism

Polymer micelles from polymers with ionizable groups can provide drug release because of a change in the pH of the environment. The pH values at which the polymer micelle breaks down and the release occurs depend on the copolymer composition. The advantage of pH-sensitive polymer micelles is that they are stable at certain pH values, but at other values, the hydrophilicity or conformation of the chains changes, causing the micelles to break down and release the substance encapsulated in them. The number of micelles that disintegrate or destabilize, and therefore the release profile, depends on the intensity of the stimulus (the degree of pH change). Once the stimulus stops, the micelles reform, and the release is interrupted [153].

**Figure 5:**
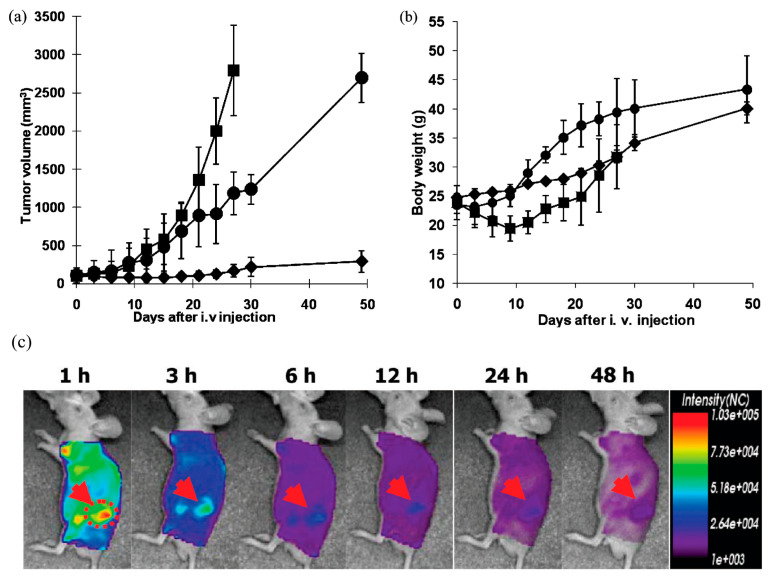
In vivo tumor growth inhibition test and body weight change in s.c. ovarian A2780/DOXR xenografted BALB/c nude mice (n = 7). A total of 10 mg/kg of DOX equivalent dose was injected as several formulations, including free DOX in PBS (■), DOX-loaded pH-insensitive micelles (DOX/PHIM-f) (●), and DOX-loaded pH-sensitive micelles (DOX/m-PHSM(20%)-f) (♦). Three IV injections on days 0, 3, and 6 were made. Values are the mean ± standard deviation (S.D.). (**a**) Tumor volume change; (**b**) body weight changes; (**c**) In vivo optical fluorescence imaging of KB tumor xenografted BALB/c nude mice after DOX encapsulated and Cy 5.5 fluorescent dye labeled pH-sensitive micelles. Arrows indicate the location of the tumor (reproduced with the permission of ACS) [163].

Poly(L-histidine) is the most commonly used pH-sensitive component in micelle-based pH-triggered release systems since this polymer contains an imidazole ring, which has a lone pair of electrons on its unsaturated nitrogen, allowing it to act as both a base (by protonation) and an acid (when already protonated). This makes the polymer amphoteric, switching its nature between hydrophobic (at neutral pH 7.4) and hydrophilic (in acidic environments) [156]. Polymeric micelles based on poly (L-histidine) that undergo destabilization in response to the acidic tumor extracellular pH were first systematically developed by Bae and co-workers [157,158,159]. These pH-responsive micelles consisting of poly(L-histidine)-b-poly(ethylene glycol) diblock copolymers (polyHis-PEG) with loaded doxorubicin (DOX) were prepared at pH 8.0 using the dialysis method and aim to exploit the protonation behavior of imidazole groups within the poly(histidine) segment [158]. In this system, the PEG block remains hydrophilic at all pH ranges, thus providing steric stabilization and prolonged circulation, while the poly(L-histidine) block exhibits pH-dependent amphiphilicity. At pH values above the pKb of poly(histidine) (≈6.5), deprotonation of the imidazole rings renders the polyHis block hydrophobic, driving the self-assembly of the copolymer into stable micelles with diameters of approximately 110 nm. When exposed to mildly acidic conditions (pH < 7.4), protonation of the imidazole groups increases the hydrophilicity of the polyHis block, leading to micelle destabilization and swelling. The destabilization induced by pH changes enables the triggered release of doxorubicin (DOX) from the micellar core. However, the release of DOX begins at pH levels slightly above the typical extracellular pH found in tumors, which indicates a significant limitation and necessitates the optimization of pH-responsive micellar systems. To enhance pH sensitivity, a mixed micelle system was developed using polyHistidine-polyethylene glycol (polyHis-PEG) block copolymers (75 wt%) and poly(L-lactic acid)-polyethylene glycol (PLLA-PEG) block copolymers (25 wt%). The micelles have an average diameter of 70 nm at pH 9.0 and are destabilized below pH 7.0. Drug release profiles showed that 32 wt%, 70 wt%, and 82 wt% of DOX were released at pH 7.0, 6.8, and 5.0, respectively, within the first 24 h. Cytotoxicity studies demonstrated that blank micelles did not exhibit significant toxicity in MCF-7 cells at concentrations up to 100 mg/mL. In contrast, DOX-loaded pH-responsive mixed micelles (PHSM) effectively killed tumor cells at pH 6.8. In vivo studies using an MCF-7 xenografted mouse model revealed that DOX-loaded PHSM (10 mg/kg) significantly inhibited tumor growth, while mice treated with free DOX experienced more weight loss compared to those treated with the PHSM formulations [160]. To facilitate the micelle formation process and to adjust the pH sensitivity, a biodegradable PLA-b-PEG-b-polyHis block copolymer was synthesized, and DOX-loaded flower-like polymer micelles were prepared. An in vitro cell cytotoxicity test conducted with the DOX-loaded PLA-b-PEG-b-polyHis micelles against MCF-7 cells demonstrated that they effectively killed the tumor cells at lower pH levels due to an increased amount of released DOX. The viability of MCF-7 cells treated with the DOX-loaded PLA-b-PEG-b-polyHis micelles was found to be 87% at pH 7.4, 40% at pH 6.8, 30% at pH 6.4, and 26% at pH 6.0 [161]. Furthermore, active targeting pH-sensitive polymer micelles were prepared by Bae et al., who developed pH-sensitive mixed micelles composed of folate-linked PEG-b-poly(L-lactide) (folate-PEG-PLLA) and PEG-b-poly(L-histidine) (PEG-polyHis) block copolymers [162]. The cytotoxicity test performed in 4T1 s.c. xenograft-bearing mice showed that DOX-loaded PEG-polyHis/folate-PEG-PLLA mixed micelles possessed significant anticancer efficacy in terms of tumor growth inhibition, improved survival, and reduced metastasis, compared to the free drug, the drug-loaded PEG-polyHis micelles, and the PEG-PLLA micelles. Additionally, the authors evaluated a second generation of pH-sensitive micelles (PHSM) composed of PEG-b-poly(L-histidine-co-L-phenylalanine) (PEG-poly(His-co-Phe)) and folate-PEG-PLLA for the delivery of doxorubicin [163]. They found that PHSM formulation resulted in a 4-fold and 10-fold increase in doxorubicin level in the tumor over PHIM and the free drug, respectively. In mice bearing multidrug-resistant ovarian A2780/DOXR s.c. xenografts, doxorubicin-loaded PHSM almost completely arrested tumor growth, whereas only modest inhibition was observed with the PHIM formulation (Figure 5). These studies strongly suggest that the combined mechanism of folate targeting and pH sensitivity of the mixed micelles contributes to enhanced drug delivery and efficacy in the tumor.

Tsai et al. reported a mixed micelle system composed of folate-PEG-PLA and poly(2-HEMA-co-histidine)-g-PLA as a pH-sensitive carrier for doxorubicin [164]. Results from the NIR imaging study indicated that the mixed micelles accumulated in the tumor to a greater extent, even though they were more rapidly eliminated from the circulation than the non-targeted micelles. In cervical adenocarcinoma HeLa s.c. xenograft-bearing mice, doxorubicin-loaded folate micelles displayed more potent tumor growth inhibition than free doxorubicin and the non-targeted micelles.

**Figure 6:**
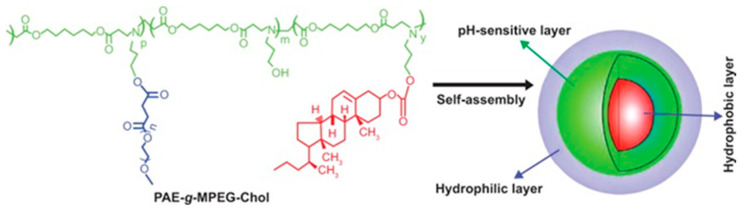
Schematic representation of micellization of poly(β-amino ester)-g-poly(ethylene glycol) methyl ether-cholesterol (PAE-g-MPEG-Chol) [169] (this work is published by Dove Medical Press Limited and licensed under Creative Commons Attribution-Non Commercial (unported, v3.0) License).

Poly(β-amino ester) (PAE) is another pH-responsive polycationic polymer used, which contains tertiary amine groups and has a pKb value around 6.5. When the pH is higher than the pKb, the PAE is deprotonated and insoluble in water, and when the pH is lower than the pKb, the PAE is able to be protonated sequentially and becomes soluble because of the ionization of the amine residues. This physicochemical property of PAE provides a significant potential in pH-triggered controlled drug release [165]. For example, amphiphilic methyl ether poly(ethylene glycol) (MPEG) and pH-responsive biodegradable poly(β-amino ester) (PAE) copolymer were used to prepare nanosized polymeric micelles, which exhibit a pH-dependent micellization/demicellization transition at tumor acidic pH 6.4, owing to the tertiary amine (pKb 6.5) in PAE. The micelles were prepared by the solvent evaporation method, and the core of the micelles was efficiently loaded (74.5%) with doxorubicin (DOX). The in vitro drug release study showed noticeable pH-dependent micellization–demicellization behavior, with rapid release of DOX from the micelles in weakly acidic environments (pH 6.4) but very slow release under physiological conditions (pH 7.4). Moreover, due to demicellization, the tumor cell uptake of DOX released from polymeric micelles was much higher at pH 6.4 than at pH 7.4. When the in vivo antitumor activity of pH-responsive polymeric micelles was evaluated by injecting the DOX-loaded polymeric micelles into B16F10 tumor-bearing mice, these micelles notably suppressed tumor growth and also prolonged the survival of the tumor-bearing mice, compared with mice treated with free DOX [166]. Similarly, camptothecin-loaded MPEG-PAE pH-responsive micelles were prepared by a solvent-casting method, and antitumor efficacy in MDA-MB-231 tumor-bearing mice was studied. The fluorescent dye tetramethylrhodamine isothiocyanate (TRITC) and the anticancer drug camptothecin (CPT) were efficiently encapsulated in the MPEG-PAE micelles, achieving a drug-loading efficiency of about 80%. The tumor-specific pH responsiveness of the MPEG-PAE micelles in MDA-MB231 human breast tumor-bearing mice was monitored via non-invasive fluorescence imaging. The encapsulated CPT exhibited effective pH-responsive release, while TRITC showed enhanced targeting to tumor tissue, being 11 times more effective than non-responsive MPEG-PLLA micelles used for comparison. Thus, prepared CPT-loaded micelles exhibited significantly increased therapeutic efficacy in breast tumor-bearing mice with minimum side effects in other tissues, compared to free CPT and CPT-encapsulated PEG-PLLA micelles [167]. Furthermore, a novel amphiphilic copolymer, poly(ethylene glycol)-b-(poly(lactic acid-co-poly(β-amino ester) (MPEG-b-(PLA-co-PAE), was synthesized, in which hydrophobic PLA and pH-sensitive PAE segments were randomly distributed within the core-forming block. Doxorubicin (DOX) was used as a model anticancer drug to prepare drug-loaded micelles. This random copolymer design was intended to uniformly distribute pH-responsive domains throughout the micellar core, thereby reducing burst drug release and enhancing drug-loading capacity. Thus, by adjusting the PAE content or environmental pH, drug release behavior can be precisely modulated. The cytotoxicity test demonstrated that the DOX-loaded micelles retained high cytotoxicity against HepG2 cells [168]. Next, an amphiphilic pH-sensitive triblock copolymer, poly(β-amino ester)-g-poly(ethylene glycol) methyl ether-cholesterol (PAE-g-MPEG-Chol), was developed to achieve controlled drug release under acidic conditions (Figure 6).

Doxorubicin (DOX) was efficiently encapsulated into the polymeric micelles with high drug-loading capacity. In vitro release studies demonstrated pronounced pH responsiveness, with slow DOX release at physiological pH (7.4), where only ~33% of the drug was released after 24 h. In contrast, at acidic pH (6.0), DOX release was markedly accelerated, with ~35% released within 3 h and nearly complete release (~95%) after 24 h. This pH-triggered behavior was attributed to protonation of amino groups in the PAE segments, leading to increased electrostatic repulsion, micelle destabilization, and enhanced drug release. Toxicity testing proved that the DOX-loaded micelles exhibited high cytotoxicity in HepG2 cells, whereas the blank copolymer micelles showed low toxicity [169]. One interesting pH-triggered micellar system was reported by Zhao et al. [170] in which Paclitaxel (PTX)-loaded mixed micelles (PTX-mM) were developed by self-assembly of stearate-modified hyaluronic acid (SHA), mPEG-b-poly(β-amino ester) (mPEG-b-PAE), and ethylene acetyl-b-poly(β-amino ester) (EA-b-PAE). During micelle fabrication, SHA micelles were sequentially coated with EA-b-PAE, followed by co-loading of PTX and mPEG-b-PAE. The resulting PTX-mixed micelles exhibited extracellular pH-triggered PEG detachment and PAE-mediated endosomal escape. Upon reducing the pH from 7.4 to 6.8, the micelle size significantly decreased from 97.5 ± 4.4 nm to 71.5 ± 2.3 nm, facilitating enhanced tumor penetration. In SKOV-3 ovarian cancer xenograft models, PTX-mM demonstrated improved tumor accumulation, a high tumor inhibition rate (64.9%), and the longest median survival time (53 days), highlighting their potential as effective therapeutics for ovarian cancer treatment [170].

Poly(2-(diisopropylamino) ethyl methacrylate (PDPA) has also been used for these purposes because they present tertiary amino groups that are also sensitive to acidic pH. pH-responsive polymeric micelles have been developed using a poly(ethylene glycol)-b-poly(2-(diisopropylamino)ethyl methacrylate) (MPEG-PDPA) block copolymer and doxorubicin (DOX) as a drug. This system offers several advantages over similar drug delivery systems, including a relatively lower critical micelle concentration (CMC), which ensures superior micellar stability under normal physiological conditions. The resulting micelles exhibit pH-triggered capabilities that allow them to transition between self-assembly and disassembly, facilitating the transport and release of the drugs in the acidic endosomal microenvironment of tumor cells and rapidly internalized by them. The cytotoxicity tests showed that the viability of the tumor HeLa breast cancer cells was significantly reduced within 3 days, which indicates that this system possesses effective anticancer efficiency [171]. To address doxorubicin (DOX) resistance in MCF-7/ADR breast cancer, a novel DOX-loaded pH-responsive micellar system was developed. This system consists of a poly(ethylene glycol)-block-poly(2-(diisopropylamino)ethyl methacrylate) (PEG-b-PDPA) diblock copolymer and a vitamin E derivative, D-α-tocopheryl polyethylene glycol 1000 succinate (TPGS), referred to as PDPA/TPGS micelles. At a neutral pH of 7.4, DOX is incorporated into the hydrophobic core of these PDPA/TPGS micelles using a film sonication method. Once inside the cells, DOX is released in early endosomes due to the acidic environment, leading to micelle dissociation. The TPGS component enhances the cytotoxic effects of DOX by targeting mitochondrial organelles and reducing the mitochondrial transmembrane potential. In vitro cell culture experiments with DOX-resistant MCF-7/ADR cells showed that PDPA/TPGS micelles reduced the IC50 of DOX six-fold. In vivo studies using animal models revealed that DOX-loaded PDPA/TPGS micelles were more effective in inhibiting tumor growth compared to free DOX in a nude mouse model bearing orthotopic MCF-7/ADR tumors. Additionally, these micelles demonstrated reduced cardiotoxicity associated with DOX, which can be attributed to the enhanced permeability and retention (EPR) effect of the micelles, resulting in a decreased distribution of DOX in the heart [172]. Furthermore, a series of ultra pH-responsive diblock copolymer micelles was prepared using a pH-dependent poly(2-(diisopropylamino) ethyl methacrylate) (PDPA) with different lengths as the core and a hydrophilic block of polyethylene glycol (MPEG) as the shell. The effects of free DOX, blank micelles, and drug-loaded micelles were tested against HeLa cells and MCF-7 cells. In vitro drug release experiments indicated that the length of the PDPA chain not only affected the drug-loading rate (which ranged from 5.4 wt% to 9.2 wt%) but also modified the drug release efficiency in acidic environments. This was attributed to the varying hydrophobicity of the PDPA blocks, which, consequently, led to reduced toxicity to normal tissues. Overall, the DOX-loaded micelles demonstrated superior anticancer efficacy, while the blank micelles exhibited minimal cytotoxicity [173].

#### 4.1.2. Polymeric Systems with Acidic pH-Induced Cleavable Bonds

Another mechanism of pH-triggered release is based on the use of pH-sensitive polymer–drug conjugates via acid-labile bonds. The most often utilized acid-labile bonds to link polymer and drug to create smart drug delivery systems that respond to endosomal/lysosomal acid pH are acetal and hydrozone bonds.

*Acetal bonds* are acid-sensitive linkages formed from a variety of hydroxyl groups, including primary, secondary, tertiary, and syn-1,2- and -1,3-diols. They are useful as acid-sensitive linkages since they rapidly hydrolyze at low pH [174]. Acetal-based delivery systems offer several advantages, including rapid degradation under endo/lysosomal pH conditions, the absence of acidic degradation products, and versatile preparation and application options. Acetal-linked polymers can be designed to create either prodrugs or prodrug-loaded micelles. The polymeric prodrugs have some disadvantages, which include complex synthesis, low drug conjugation, small size, and reduced drug effectiveness due to slow drug release at the site of action and/or chemical alteration of the released drug [175]. To combine the advantageous features of polymeric prodrugs and micellar nanoparticles, the development of prodrug micelle systems is gaining increased attention. For example, novel endosomal pH-responsive paclitaxel (PTX) prodrug micelles with high drug content of 23.5 wt% have been developed using a functionalized poly(ethylene glycol)-poly(ε-caprolactone) (mPEG-PCL) diblock polymer. The acetal linker is formed between the 2′-OH group of paclitaxel and the pendant vinyl ethers of PCL obtained after chemical modification. Cytotoxicity tests demonstrated that these micelles have a higher therapeutic efficacy against MCF-7 cells compared to free PTX [176]. In another study, endosomal pH-responsive paclitaxel (PTX) prodrug micellar nanoparticles were prepared by conjugating PTX to water-soluble poly(ethylene glycol)-b-poly(acrylic acid) (PEG-PAA) block copolymers via an acetal bond to the PAA block, using ethyl glycol vinyl ether (EGVE) as a linker. In vitro release studies indicated that drug release from the PTX prodrug nanoparticles is highly pH-dependent and shows a significant antitumor effect against KB and HeLa cells, as well as PTX-resistant A549 cells. Notably, folate-decorated PTX prodrug micellar nanoparticles, based on a PTX-loaded PEG-PAA system and 20 wt% folate-poly(ethylene glycol)-b-poly(d,l-lactide) (FA-PEG-PLA), displayed a marked ability to target folate receptor-overexpressing KB cells. The IC50 value for these nanoparticles was over 12 times lower than that of non-targeting PTX-loaded PEG-PAA under identical conditions [175]. A potential strategy to combat drug resistance in cancer therapy involves the mitochondrial targeting of drugs by attaching a lipophilic cation such as (3-carboxypropyl) triphenylphosphonium bromide (TPP) to existing medications [177,178]. Based on this strategy, an amphiphilic block copolymer was prepared by attaching DOX-TPP conjugate to acetal-functionalized PEG-PCLL block copolymer. This led to the formation of DOX-TPP-loaded acetal-PEG-PCLL micelles. The cytotoxicity of these micelles was studied against MCF-7 cells and MCF-7/ADR cells. The results showed better antitumor efficacy compared to free doxorubicin in MCF-7/ADR cells. Additionally, the apoptotic rate and cellular uptake of the micelles were significantly higher than those of free DOX and DOX-TPP conjugate. These findings conclude that the micelles efficiently deliver mitochondrial-targeting DOX-TPP to tumor cells [179].

*Hydrazone bonds* are other acid-labile linkages that remain relatively stable at physiological pH (~7.4) but are cleaved quickly in acidic environments such as the tumor extracellular pH (~6.5–6.8) or the endosomal/lysosomal pH (~5–6) inside cells. When drugs are conjugated to polymers via hydrazone linkers, the resulting micelles can release the drug preferentially under acidic conditions, improving tumor specificity and reducing systemic toxicity. Hydrazone linkers have been widely used in polymeric micelle platforms to achieve pH-responsive drug release in cancer therapy. Doxorubicin is the most frequently employed example used for hydrazone-linked polymeric micelles due to its favorable chemical structure. The pioneering work was first reported by Park et al. and Kataoka et al. For instance, doxorubicin was chemically conjugated to the terminal end of a diblock copolymer composed of poly(l-lactic acid) (PLLA) and methoxy-poly(ethylene glycol) (mPEG) via two acid-cleavable linkages: a hydrazine and cis-acotinyl bond between doxorubicin and the terminal group of PLLA segment in the block copolymer. Doxorubicin-conjugated PLLA-mPEG diblock copolymers self-assembled to form micelles in aqueous solution. It was estimated that in an acidic condition, the conjugated doxorubicin via hydrazone linkage was easily cleaved, releasing doxorubicin in an intact structure. Doxorubicin-conjugated PLLA-mPEG micelles were more potent in cell cytotoxicity than free doxorubicin, suggesting that they were more easily taken up within cells with concomitant rapid release of cleaved doxorubicin into the cytoplasm from acidic endosomes [180]. In another study, a poly (ethylene glycol)-poly(b-benzyl-l-aspartate) (PEG-PBLA) was used as a convenient template, which was further modified to prepare PEG-p(Asp-Hyd) block. The DOX molecules were then conjugated to the polymer backbone through an acid-labile hydrazone bond [181]. In addition, DOX was conjugated via hydrazone linkage onto biodegradable block copolymer based on poly(ethylene) glycol-b-poly(lactide-co-2,2-dihyroxymethylpropylene carbonate) (mPEG-b-P(LA-co-DHP). Mixed micelles consisting of mPEG-b-P(LA-coDHP/DOX) and folic acid-decorated (mPEG-b-P(LA-co-DHP) were prepared and tested against the human ovarian cancer cell line SKOV-3. The results demonstrated preferential internalization into the cancer cells of FA-containing micelles in comparison to those without FA [182]. Recently, the biopharmaceutical company NanoCarrier (Chiba, Japan) developed a micellar system known as NC-6300. This system consists of epirubicin (EPI) covalently bonded to a PEG polyaspartate block copolymer through an acid-labile hydrazone bond. In vitro studies indicated that NC-6300 exhibited pH-dependent release of EPI, which accelerated under increasingly acidic conditions. NC-6300 is stable in the bloodstream for an extended period, allowing it to selectively accumulate in tumor tissues due to the enhanced permeability and retention effect [183]. The antitumor effect of NC-6300 using mouse models with human hepatocellular carcinoma Hep3B cells was evaluated. The results showed that NC-6300 had a significantly stronger antitumor effect against subcutaneous tumors of Hep3B compared to epirubicin. Additionally, NC-6300 resulted in a much longer survival rate than epirubicin in mice with liver orthotopic tumors of Hep3B. In terms of cardiotoxicity, mice treated with epirubicin exhibited significant deterioration in fractional shortening and ejection fraction [184]. In addition to doxorubicin, hydrazone-based prodrug polymeric micelles have been developed for several clinically relevant anticancer agents, including paclitaxel and platinum (IV) prodrugs. Early prodrug micelles, which utilize PEG-block-poly(aspartate-hydrazide) for conjugation, showed that when paclitaxel is linked via levulinic acid or 4-acetyl benzoic acid, it releases significantly faster at an acidic pH compared to physiological pH. This finding indicates enhanced specificity for tumors and a higher cytotoxic effect against SK-OV-3 and MCF-7 cancer cell lines [185]. In addition, hydrazone-linked mPEG-cholesterol-chitosan paclitaxel prodrug micelles showed stability at pH 7.4 and rapid PTX release at pH 5.0, with improved cytotoxicity and antitumor efficacy in vivo [186]. Platinum-based hydrazone prodrug micelles, especially those that incorporate Pt(IV) complexes conjugated to amphiphilic polymers, expand the potential uses of this strategy. They enable the intracellular reduction and pH-triggered activation of platinum drugs, improving their therapeutic efficacy while reducing systemic toxicity. A newly reported drug delivery vehicle for cisplatin is the acid-responsive polymer–platinum conjugate 46. This nanoparticulate system is created by covalently linking the platinum (IV) prodrug to the hydrophobic segment of two biocompatible diblock copolymer chains through a pH-sensitive hydrazone bond. This system exhibits a highly differential drug release profile depending on environmental acidity. In the bloodstream, where the pH is nearly neutral (7.4), the drug remains contained. However, upon entering cancer cells via endocytosis, the acidic intracellular pH (approximately 5.6) triggers a rapid release of the drug. This swift release of high doses of drugs within cancer cells can help overcome chemoresistance and improve the therapeutic effectiveness of the treatment. The conjugate demonstrates a well-controlled platinum loading yield, excellent drug release kinetics, and enhanced in vitro cytotoxicity against A2780 ovarian cancer cells compared to free cisplatin [94].

### 4.2. Thermosensitive PEG-Based Polymer Micelles

Another group of widely studied intelligent polymer systems is those using thermosensitive polymers (TRPs), which undergo a reversible phase transition in response to a temperature change. Based on their reaction, these polymers are categorized into two classes: (i) Polymers that become insoluble above a certain critical temperature, known as the lower critical solution temperature (LCST). They are completely soluble in aqueous solutions under normal temperature conditions, but as the temperature rises above the critical value, their solubility decreases, and they exhibit phase separation. (ii) Polymers that precipitate and undergo a phase change below a certain critical temperature, known as the upper critical solution temperature (UCST). This is the temperature above which these polymers remain miscible in the solution, and when the temperature of the solution falls below the critical value, phase separation occurs [187].

Thermo-sensitive polymeric micelles represent an important class of stimuli-responsive drug delivery systems, capable of reversibly forming core–shell nanostructures in response to temperature changes. The temperature-induced transition of the thermosensitive block enables efficient encapsulation of hydrophobic drugs and controlled release under mild hyperthermia conditions. Owing to these properties, such micelles have attracted significant attention for biomedical and anticancer applications [188]. Poly(N-isopropylacrylamide) (PNIPAM) and PEO-b-PPO-b-PEO block copolymers, known as Pluronics, remain the most widely investigated thermosensitive materials for anticancer drug delivery due to their well-defined thermal transitions, ease of formulation, and extensive preclinical validation [187,188,189,190].

Poly(N-isopropylacrylamide) (pNIPAAm) is a thermosensitive polymer, which possesses an LCST around 32 °C [191]. Below this temperature, it is water-soluble (hydrophilic), and above this temperature, it is water-insoluble (hydrophobic/collapsed). This property makes it a good choice as a constructive block for polymer micelle formation, which, depending on the LCST, can form micelles with a pNIPAAm shell or pNIPAAm core, respectively. Copolymers of pNIPAAm with permanently hydrophobic blocks such as poly(d,l lactide) [192], poly(methylmethacrylate) [193], and poly(butylmethacrylate) [194] form micelles with a hydrated pNIPAAm corona below its LCST. When heated above the LCST, the micellar shell becomes hydrophobic, leading to micelle destabilization and aggregation. However, pNIPAAm is not suitable for in vivo applications as its LCST is below body temperature, and the micelles with pNIPAAm as a shell are expected to rapidly aggregate upon injection [195].

The LCST of PNIPAAm can be optimized by random copolymerization with other monomers in order to achieve improved targeting and drug release. By copolymerizing with more hydrophilic monomers, the LCST of PNIPAAm can be increased, making it suitable as a hydrophilic segment for heat-triggered release. Three monomers that have been investigated in combination with PNIPAAm are acrylamide (AAm), hydroxymethylacrylamide (HMAAm), and dimethylacrylamide (DMAAm). It was found that copolymers of PNIPAAm with these hydrophilic monomers can have an LCST above body temperature [194]. Various preclinical studies have evaluated thermosensitive micelles based on these copolymers for encapsulating doxorubicin or paclitaxel, and all of them have shown significantly enhanced cytotoxic effects at temperatures above normal physiological conditions [196,197,198].

PNIPAAm can also be used as a core-forming block of micelles since it is hydrophobic at body temperature. As a hydrophobic fragment, PNIPAAm is often conjugated to PEG to form stable micelles. The micelles can be prepared by heating the solution above the LCST of PNIPAAm [199]. Micelles formed by PEG-PNIPAM copolymers have been explored for encapsulating lipophilic drugs, with temperature-triggered disassembly enabling controlled drug release. These micelles possess a hydrophilic PEG shell and a thermosensitive PNIPAM core. The block copolymer composition, including block length and the hydrophilic/hydrophobic ratio, is crucial for optimizing polymeric micelles in drug delivery [200]. Currently, there is no available literature on drug loading in polymer micelles made from linear PEG-b-PNIPAM block copolymers. However, a multifunctional micellar drug carrier has been designed and prepared using a thermosensitive and biotinylated double-hydrophilic block copolymer (DHBC), specifically biotin-poly(ethylene glycol)-block-poly(N-isopropylacrylamide-co-N-hydroxymethylacrylamide) (biotin-PEG-b-P(NIPAAm-co-HMAAm)). By adjusting the feed ratio of the P(NIPAAm-co-HMAAm) block, a reversible phase transition at a lower critical solution temperature (LCST) of 36.7 °C can be achieved. Cytotoxicity studies show that the blank biotin-PEG-b-P(NIPAAm-co-HMAAm) copolymer exhibits no significant cytotoxic effects. Furthermore, the micelles were loaded with an anticancer drug, methotrexate (MTX), and the in vitro release behavior of the obtained micelles at different temperatures was investigated. The results demonstrated that DHBC drug carriers can specifically and effectively bind to cancer cells when pretreatment with biotin-transferrin is applied, suggesting that they may serve as a valuable drug carrier for tumor targeting [201].

**Figure 7:**
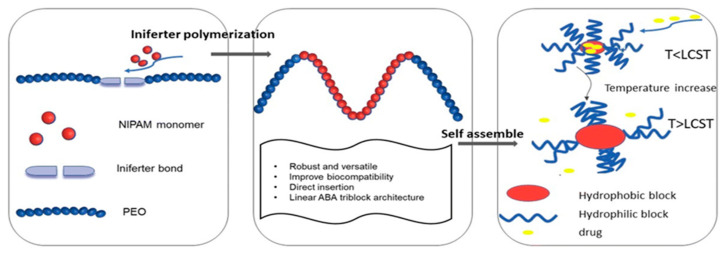
Illustration of preparing PEO-PNIPAM-PEO block copolymers and drug delivery application [202] (this article is licensed under a Creative Commons Attribution-NonCommercial 3.0 Unported License).

Using a PEGylated macroiniferter, a PEO–PNIPAM–PEO triblock copolymer was synthesized, which subsequently self-assembled into thermoresponsive micelles with a lower critical solution temperature (LCST) of approximately 36 °C, making them suitable for temperature-triggered drug delivery in cancer therapy. These micelles were used to encapsulate the anticancer drug doxorubicin (DOX) (Figure 7). In vitro studies demonstrated that the polymeric micelles were biocompatible and were more efficiently internalized by HeLa cells than free DOX at equivalent concentrations. Notably, the micellar system exhibited significantly enhanced cumulative DOX release at elevated temperatures, consistent with its thermosensitive behavior. Furthermore, cellular uptake and viability assays confirmed that DOX-loaded micelles produced superior therapeutic efficacy compared with free DOX at the same dose, highlighting their potential as effective thermoresponsive drug carriers [202].

Thermosensitive polymeric micelles based on comb-like copolymers composed of methoxy poly(ethylene glycol) (mPEG) blocks and hydrophobic polyacrylate (PA) backbones grafted with poly(N-isopropylacrylamide) (PNIPAM) chains (mPEG-b-PA-g-PNIPAM) were also developed and loaded with camptothecin (CPT). Key parameters, including incubation temperature, drug loading, and copolymer composition, particularly the molecular weight of the mPEG and PNIPAM segments, were systematically optimized. CPT release was significantly accelerated when the environmental temperature exceeded the lower critical solution temperature (LCST), demonstrating effective thermo-triggered drug release behavior. In vitro cytotoxicity studies conducted in MDA-MB-231 human breast cancer cells showed that CPT-loaded micelles exhibited slightly lower cytotoxicity than free CPT at equivalent drug concentrations, which was attributed to the sustained and time-dependent release of CPT from the micellar carriers [203].

Thermoresponsive poly(ethylene oxide)-poly(propylene oxide)-poly(ethylene oxide) (PEO-PPO-PEO), known as Pluronics, were also extensively studied as potential drug carriers. By adjusting the concentration, composition, and molecular weight, these copolymers can be tuned to undergo reversible phase transitions at physiological temperature and pH. The combination of hydrophilic ethylene oxide and hydrophobic propylene oxide units results in an amphiphilic copolymer that, above the critical micelle concentration (CMC) in aqueous conditions, self-assembles into micelles. The CMC is highly temperature-dependent, as below the critical micelle temperature (CMT), the ethylene oxide and propylene oxide blocks are relatively soluble in water. As the temperature of the system increases, the polypropylene oxide chain becomes less soluble, leading to the formation of micelles. These micelles typically have a diameter ranging from 10 to 100 nm and consist of a hydrophobic core composed of polypropylene oxide and a hydrated, hydrophilic shell made of polyethylene oxide [204,205,206]. The biological activity of Pluronic micelles is primarily attributed to their ability to insert into cellular membranes and subsequently translocate into cells, where they modulate multiple cellular processes. These include mitochondrial respiration, ATP production, the activity of drug efflux transporters, apoptotic signaling pathways, and gene expression. Consequently, Pluronics markedly sensitize multidrug-resistant (MDR) tumors to a wide range of anticancer agents, enhance drug transport across the blood–brain and intestinal barriers, and induce transcriptional activation of gene expression both in vitro and in vivo [204]. A number of studies demonstrated the loading of different anticancer drugs, such as doxorubicin and paclitaxel, into Pluronics-based micelles, which possess enhanced anticancer activity in in vivo models [207,208,209,210]. In these studies, mice bearing drug-sensitive and drug-resistant tumors were treated, which included murine leukemia, murine myelomas, Lewis lung carcinoma, human breast carcinomas, and human oral epidermoid carcinoma. SP1049C is the first anticancer micellar formulation reaching clinical evaluation and represents a product that contains doxorubicin (Dox) in the mixed micellar system consisting of Pluronics L61 and F127 [211]. This formulation demonstrated better in vivo antitumor activity than DOX in both drug-resistant and drug-sensitive tumor models, as well as improved pharmacokinetics and tumor accumulation [207]. The higher activity of SP1049C compared with DOX was shown to be due to increased cellular uptake and inhibition of the drug efflux, as well as changes in the intracellular drug tracking. The formulation showed promising results, including slower clearance than conventional DOX, as well as evidence of antitumor activity in some patients with advanced, resistant solid tumors. SP1049C proceeded in a phase II study in patients with advanced adenocarcinoma, and the formulation showed notable single-agent activity and an acceptable safety profile [207]. Furthermore, several polymer-mixed Pluronic-based micelles loaded with anticancer drugs have been developed. For example, polymeric mixed micelles composed of Pluronic P105 and F127 were prepared to deliver the poorly soluble anticancer drug docetaxel (DTX) for the treatment of Taxol^®^-resistant non-small cell lung cancer. In vitro studies showed comparable IC_50_ values for Taxotere^®^ and DTX-loaded micelles in A549 cells; however, in resistant A549/Taxol cells, the mixed micelles exhibited a pronounced hypersensitization effect, reducing the IC_50_ to 0.059 µg/mL compared with 0.593 µg/mL for Taxotere^®^. Pharmacokinetic evaluation demonstrated prolonged systemic circulation, with a 1.85-fold increase in mean residence time and a 3.82-fold higher AUC relative to Taxotere^®^. Consistently, in vivo antitumor studies revealed a significantly higher tumor inhibition rate for the mixed micelles (69.05%) compared to Taxotere^®^ (34.43%; *p* < 0.01), highlighting their potential to overcome multidrug resistance in lung cancer [212]. Dual drug-loaded mixed micelles were also developed as Pluronic P105-DOX conjugates, which represent hydrophobic cores to co-encapsulate paclitaxel (PTX) with Pluronic F127, forming dual drug-loaded mixed micelles (PF–DP) capable of co-delivering hydrophilic DOX and hydrophobic PTX. Cellular uptake studies demonstrated efficient accumulation of both agents in multidrug-resistant (MDR) cancer cells. In vitro cytotoxicity, apoptosis, and cell cycle assays showed superior antitumor efficacy of PF-DP compared with single-drug micelles, indicating synergistic effects. Furthermore, in MCF-7/ADR tumor-bearing mice, PF-DP treatment produced significantly stronger tumor suppression than combined free DOX and PTX administration. These findings suggest that dual drug-loaded Pluronic-based mixed micelles are a promising nanocarrier for MDR cancer chemotherapy [213].

**Figure 8:**
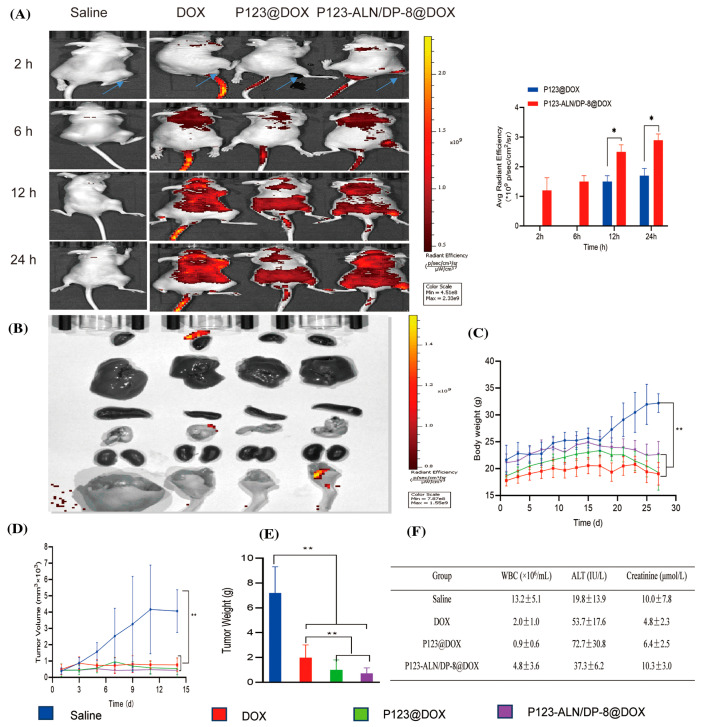
In vivo biodistribution and therapeutic efficacy in a murine breast cancer bone metastasis model. Saline (control), free DOX, P123@DOX micelles, and P123-ALN/DP-8@DOX micelles were intravenously administered on days 14 and 21 (DOX dosage of 5 mg/kg). (**A**) Representative whole-body fluorescence images of tumor-bearing mice 2, 6, 12, and 24 h after intravenous injection of treatments. (**B**) Representative fluorescence images of the organs at the end of the experiment. (**C**) Body weight of mice as a function of time after intratibial MDA-MB-231 cell inoculation and therapy. (**D**) Tumor volume. (**E**) Tumor weight at the end of the experiment. (**F**) Blood chemistry at the end of the experiment. Data are expressed as the mean ± SD (n = 6). * *p* < 0.05, and ** *p* < 0.01 [216] (this work is licensed under a Creative Commons Attribution 4.0 International License).

In addition, folic acid-functionalized Pluronic P123/F127 mixed micelles encapsulating paclitaxel (FPF-PTX) were developed and evaluated versus non-targeted micelles (PF-PTX) and Taxol^®^. Cellular uptake studies demonstrated significantly enhanced internalization of FPF-PTX. It was shown that after incubation for 2 h at 37 °C, the uptake of FPF-PTX by folate receptor over-expressing cells (KBv and KB cells) was much higher than that of PF-PTX (*p* < 0.05). On the contrary, for folate receptor-deficient cells (A-549 cells), no difference was observed between PF-PTX and FPF-PTX, indicating that folic acid-modified polymeric micelles could enter the cells via the receptor-mediated pathway. Consistently, in vitro cytotoxicity, apoptosis induction, and cell cycle arrest assays showed superior anticancer activity of FPF-PTX relative to PF-PTX and Taxol^®^. Pharmacokinetic studies in rats revealed approximately threefold higher bioavailability of PTX from micelles compared to Taxol^®^. Furthermore, in BALB/c mice bearing KBv multidrug-resistant tumor xenografts, FPF-PTX exhibited markedly enhanced antitumor efficacy, attributed to the combined effects of folate-mediated targeting and the MDR-reversal capability of Pluronic copolymers [214]. In another approach, Guo et al. developed FA-decorated Pluronic F127-PLA (FP)-based thermosensitive micelles with active tumor-targeting capability. These amphiphilic micelles were efficiently internalized by folate receptor-overexpressing tumor cells via receptor-mediated endocytosis and enabled rapid intracellular drug release under mild hyperthermia (40 °C). Micelles containing a PLA segment (DP = 100) exhibited a lower critical solution temperature (LCST) of 39.2 °C, making them suitable for near-physiological applications. DOX release from these micelles was minimal at 37 °C but markedly accelerated at 40 °C due to thermally induced micelle shrinkage. This temperature-responsive behavior allowed stable drug retention under normal physiological conditions and rapid release upon mild hyperthermia, highlighting the potential of the micelles for thermally triggered, targeted cancer therapy [215]. Recently, dually decorated Pluronic P123 (P123)-based polymeric micelles functionalized with alendronate (ALN) and a breast cancer-specific phage protein (DMPGTVLP, DP-8) were developed for targeted therapy of breast cancer bone metastases. Doxorubicin (DOX) was encapsulated into the micelle core with a high loading capacity (3.44%), forming spherical micelles (~123 nm) with a narrow size distribution. In vitro, DOX release was accelerated under acidic conditions (pH 5.0), and the micelles were efficiently internalized by breast cancer cells, inducing higher cytotoxicity than free DOX. The micelles demonstrated strong hydroxyapatite binding, indicating high bone affinity. In a 3D cancer bone metastasis model, P123-ALN/DP-8@DOX inhibited tumor growth and reduced bone resorption. In vivo studies on a mouse model with breast cancer bone metastasis showed enhanced tumor accumulation, significant antitumor activity, and minimal systemic toxicity (Figure 8). These results suggest that pH-sensitive, dual-ligand-targeted polymeric micelles are a promising strategy for breast cancer bone metastasis treatment [216].

### 4.3. Multi-Responsive PEG-Based Polymer Micelles

To enhance the therapeutic effectiveness of encapsulated chemotherapeutic drugs within polymer micelles, it is important to optimize and control the drug release process. This can be achieved by developing polymer micelles that respond to multiple stimuli, such as pH, temperature, redox potential, and light, among others. The advantage of these systems is that responses can occur either simultaneously at the same location or in a sequential manner in different settings and/or compartments. These dual and multi-stimuli-responsive polymeric nanoparticles could provide excellent control over drug delivery and release, resulting in improved anticancer effectiveness both in vitro and in vivo [217].

*Dual pH/redox-responsive PMs*

Redox and pH dual-responsive drug delivery micelles have gained significant attention in recent years for cancer treatment, due to the differences in redox potential and pH gradient that exist in healthy and cancer cells. Glutathione (GHT) is a natural reducing agent for disulfide bonds with a blood plasma level of approximately ~2 µM, due to enzymatic degradation, while the intracellular concentration of GSH in cancer cells is several times higher than that in normal cells and ranges from 2 to 10 mM [218]. In addition, the pH level at both primary and metastasized tumors is lower (6.5–7.2) than the pH of normal tissues (7.4), and the intracellular pH of their endosomes (5.5–6.0) and lysosomes (4.5–5.0) is even more acidic [219]. These significant differences in pH and redox potential can selectively trigger the destabilization of the micelles in tumor tissue, which occurs due to pH sensitivity and the reduction (cleavage) of disulfide bonds. Therefore, pH and redox dual-sensitive nanoparticles have been designed and developed to trigger drug release or enhance tumor cell uptake via the differences in tumor pH, and to accelerate drug release in a controlled manner in the endo/lysosomal compartments and/or in the cytoplasm and nucleus of cancer cells.

For example, dual-sensitive pH/redox polymer micelles were developed using poly(ethylene glycol) and biodegradable polycarbonate, bearing a glutathione (GSH)-sensitive disulfide bond and pH-responsive carboxylic acid groups. Doxorubicin (DOX) was loaded into these micelles through ionic interactions. In vitro studies demonstrated enhanced cytotoxicity against the BT-474 human breast cancer cell line. Additionally, in vivo experiments conducted with nude mice bearing BT-474 xenografts showed that the DOX-loaded micelles exhibited excellent antitumor efficacy. Importantly, these micelles caused negligible toxicity to the mice due to the effective delivery of DOX. In contrast, mice treated with free DOX demonstrated substantial body weight loss and cardiotoxicity [220]. Novel dual-redox and pH-responsive micelles based on poly(ethylene glycol)-SS-poly(2,4,6-trimethoxybenzylidene-pentaerythritol carbonate) (PEG-SS-PTMBPEC) copolymer with encapsulated doxorubicin (DOX) were prepared and investigated for intracellular doxorubicin (DOX) release. It was proposed that upon endocytosis, the DOX-loaded PEG-SS-PTMBPEC micelles would release some of the DOX in the mildly acidic environment of endosomal compartments due to the hydrolysis of acetal bonds. After the micelles escape from the endosomes, the cleavage of disulfide bonds triggered by the high concentration of glutathione (GSH) in the cytosol will facilitate a more complete release of DOX. Indeed, the drug release profile indicates that the release of doxorubicin (DOX) was significantly accelerated at a pH of 5.0 or in the presence of 10 mM glutathione (GSH) at pH 7.4. Under these conditions, 62.8% and 74.3% of DOX were estimated to be released within 21 h, respectively. By increasing the GSH concentration to 10 mM and lowering the pH to 5.0, drug release was further enhanced, achieving 94.2% release within just 10 h. It was found that DOX was delivered and released into the nuclei of HeLa cells, while in the reduction, insensitive PEG-PTMBPEC control DOX was mainly located in the cytoplasm. MTT assays revealed that DOX-loaded PEG-SS-PTMBPEC micelles exhibit higher antitumor activity than reduction-insensitive controls, thereby making them a promising platform for targeted intracellular anticancer drug release [221].

In another example, dual-sensitive biodegradable polypeptide micelles with encapsulated doxorubicin, which can respond to redox and pH environments, were also developed using methoxy poly(ethylene glycol)-b-poly [2-(dibutylamino)ethylamine-L-glutamate] (mPEG-SS-PNLG) copolymer. The drug release study of the micelles performed at pH 7.4 indicates a relatively low percentage of drug release within 24 h compared to the release profile at pH 5.0, where significantly accelerated drug release was observed. DOX-loaded mPEG-SS-PNLG micelles significantly increased in vivo therapeutic efficacy toward HepG2 cells compared to the free-DOX and control groups, making them promising candidates for a drug delivery system [222]. Furthermore, a dual pH- and redox-responsive drug delivery system was developed using methoxy-poly(ethylene glycol) (mPEG) and polysuccinimide (PSI) polymers connected via disulfide linkages, followed by modification of the PSI segment with 2-diisopropylaminoethylamine (DIPEA) and hydrazine hydrate (Hy). In this system, DOX was chemically conjugated through acid-labile hydrazone bonds, while the free DOX molecules were physically encapsulated via hydrophobic interactions and π–π stacking between the aromatic rings, resulting in the formation of DOX-loaded micelles. The release profiles study indicated that the DOX-loaded micelles showed a pH and reduction-dependent responsiveness, as the cumulative drug release percentage of DOX-loaded micelles at pH 5.0 with 10 mM GSH was higher than that at pH 5.0 without GSH or at pH 7.4 with 10 mM GSH. Enhanced cytotoxicity of DOX-loaded micelles against adenocarcinomic human alveolar basal epithelial cells (A549 cells) in response to changes in pH or GSH concentration was determined [223].

Mixed micelles with dual pH- and redox-responsiveness were also prepared using pH-responsive poly(ethylene glycol) methyl ether-b-poly(β-amino esters) copolymer (mPEG-b-PAE) and redox-responsive poly(ethylene glycol) methyl ether-grafted disulfide-poly(β-amino esters) copolymer (PAE-ss-mPEG). To study the drug delivery properties of the system, doxorubicin (DOX) was encapsulated into micelles. In vitro drug release profiles of the DOX-loaded mixed micelles at different pH and DL-dithiothreitol (DTT) content demonstrated controlled release depending on the pH and DTT. The cytotoxicity assay confirms that DOX-loaded mixed micelles are able to inhibit the HepG2 cell growth. These results suggested that the dual pH- and redox-responsive polymeric micelles self-assembled from two or more types of stimuli-responsive copolymers could be an effective method to prepare multifunctional drug delivery systems [224].

Next, mixed polyprodrug micelles (MPPMs) with pH/redox dual-triggered drug release property were developed using two polyprodrugs. The first one consists of a methyl ether poly(ethylene glycol)-b-poly(β-amino esters) copolymer conjugated with DOX through a pH-sensitive acid-labile cis-aconityl bond (mPEG-b-PAE-cis-DOX), and the second one consists of a methyl ether poly(ethylene glycol)-b-poly(β-amino esters) copolymer conjugated with DOX through a GSH-sensitive disulfide bond (mPEG-b-PAE-ss-DOX). The in vitro drug release profiles of DOX-loaded MPPMs demonstrated pH/redox dependence properties. It was found that MPPMs could efficiently deliver the DOX molecules to the tumor cell nucleus compared to the free DOX. In vivo, DOX-loaded micelles demonstrated enhanced antitumor activity and effectively inhibited tumor growth with improved therapeutic efficacy and reduced side effects [225].

Recently, block copolymers based on alpha-lipoic acid (αLA) and methoxy poly(ethylene glycol) (mPEG-PαLA) were synthesized. These amphiphilic mPEG-PαLA copolymers effectively encapsulate both paclitaxel (PTX) and doxorubicin (DOX) during their self-assembly into micelles in aqueous solutions. This process generates nanoparticles that co-load PTX and DOX for simultaneous delivery in osteosarcoma therapy. The dual-drug-loaded nanoparticles were efficiently internalized by K7 osteosarcoma cells and released the drugs intracellularly. The PTX-DOX-loaded micelles demonstrated synergistic therapeutic effects, leading to increased cell apoptosis in K7 cells. These nanoparticles also exhibited improved biodistribution and greater efficacy in inhibiting tumor growth compared to control groups in a murine model of osteosarcoma [226]. In addition, dual pH/redox responsive nanomicelles containing the anticancer drug doxorubicin (DOX) are formed through the self-assembly of a triphenylphosphonium-grafted PEG-poly(D, L-lactide) copolymer with a disulfide linkage between its blocks, aiming for targeted delivery to mitochondria in cells. To enhance blood circulation and improve tumor cell endocytosis, the surface positive charges of TPP are converted to negative charges by coating the nanoparticles with chondroitin sulfate (CS). CD44, a glycoprotein overexpressed in various cancers, interacts with the CS layer to mediate selective endocytosis of the nanoparticles, facilitating targeted drug delivery. CS is stable and hydrophilic at pH 7.4 but becomes hydrophobic at pH 5.5. In the acidic environment of lysosomes, the CS layer is removed, exposing TPP and reversing the nanoparticles’ charge. These nanoparticles then attach to the mitochondrial outer membrane, leading to depolarization of the membrane and increased permeability. This results in the overproduction of reactive oxygen species (ROS), promoting apoptosis. Released DOX, following nanoparticle disassembly triggered by glutathione (GSH), diffuses directly into the mitochondria due to enhanced permeability. The synergistic effects of the micelle showed greater cytotoxicity [227].

A novel active targeting folate-decorated redox and pH dual-responsive micellar drug delivery system was developed based on folate-poly(ethylene glycol)-b-poly((α-paclitaxel-SS-caprolactone)-co-caprolactone) (FA-PEG-b-P((PTX-SS-CL)-co-CL)) conjugates with thiol and acid-cleavable linkages. In vitro drug release studies demonstrated that the prodrug micelles are relatively stable at normal physiologic conditions, but they are pH- and redox-dependent at conditions that mimic the tumor environment, leading to pronounced PTX release. Folate-decorated prodrug micelles were selectively taken up by HeLa tumor cells via FA-receptor-mediated endocytosis. The MTT test showed that the therapeutic efficacy of these micelles against HeLa cancer cells was enhanced compared to free PTX [219].

*Dual pH/thermoresponsive micelles*

Dual-stimuli-responsive polymer micelles that are sensitive to pH and temperature are another promising approach for cancer therapy. This is due to the acidic environment of tumors and the relatively higher local temperatures, which can be enhanced by applied external heat sources, allowing for better targeting and treatment of cancerous tissues. These systems can be highly effective because they can make precise differentiation between pathological and healthy tissues.

For example, a dual-responsive mixed micellar system with encapsulated DOX that is responsive to temperature and pH was developed using methoxy-PEG-b-P(N-(2-hydroxypropyl) methacrylamide dilactate)-co-(N-(2-hydroxypropyl) methacrylamide-co-histidine) (mPEG-b-P(HPMA-Lac-coHis) and mPEG-b-PLA copolymers. The cytotoxicity activity was evaluated in vitro against different cell lines, including HeLa, ZR-75-1, MCF-7, and H661. The micelles displayed a high cytotoxic effectiveness at pH 5.4 and 37 °C, which is similar to the acidic endosomal compartment in comparison to free DOX. It was found that Dox-micelles could effectively release the drug into the cell nuclei in a more controlled manner, particularly in the acidic endocytic compartments. In vivo study on Balb-c/nude mice bearing human cervical cells showed that dual-responsive micelles exhibited considerably higher antitumor activity compared to free doxorubicin, while also reducing the drug’s toxicity to normal tissues. The results of this study suggest that biodegradable dual-responsive particles exhibit a specific targeting efficiency and an excellent antitumor activity [228]. The high antitumor activity of DOX-loaded dual-responsive nanoparticles was attributed to their tumor-specific accumulation, enhanced permeation through the tumor site, and tumor pH-triggered drug release.

In a more recent example, dual-responsive pH and temperature hybrid micelles with a double-locked drug delivery system were developed. For this purpose, temperature-sensitive polyethylene glycol-poly(tetrahydropyranylmethacrylate)-polyethylene glycol (PEG–PTHPMA–PEG) copolymer synthesized by RAFT polymerization was combined with pH-sensitive diblock polymers, poly(2-(diisopropylamino ethylmethacrylate)-polyethylene glycol (PDPA-PEG). These copolymers self-assemble in aqueous solutions to form hybrid micelles with encapsulated doxorubicin (DOX). The release of the drug occurs with the simultaneous stimulation of both triggers: pH and temperature. It was found that the micelles maintain their structural stability when exposed to a single stimulus. However, under dual stimulation, they collapse and recombine, resulting in significant drug release, as demonstrated by the high cytotoxicity effect. The dual-responsive hybrid micelles provide a double insurance mechanism against drug leakage and allow precise control over drug release, which makes them promising candidates for accurate drug delivery systems [229].

A series of novel thermo- and pH-responsive block copolymers, specifically PHis-PLGA-PEG-PLGA-PHis, were synthesized. These copolymers consist of poly(ethylene glycol) (PEG), poly(D,L-lactide-co-glycolide) (PLGA), and poly(L-histidine) (PHis) and were utilized to construct stimuli-responsive copolymer micelles. The starting polymers, PLGA-PEG-PLGA and PHis, were synthesized through the ring-opening polymerization of DL-lactide and glycolide, using PEG as an initiator, and from L-histidine N-carboxyanhydride with isopropylamine as an initiator, respectively. The final copolymer was obtained by the coupling reaction of PHis with PLGA-PEG-PLGA. The resulting copolymer micelles were designed to have an inner core composed of two hydrophobic blocks (PLGA and deprotonated PHis) and an outer hydrophilic PEG shell. In vitro studies confirmed the temperature- and pH-responsive properties of the copolymer micelles through drug release, cytotoxicity, and intracellular localization assessments. The in vitro cytotoxicity of the doxorubicin (DOX)-loaded micelles was evaluated on MCF-7 human breast cancer cells using the MTT assay, with blank micelles and DOX solution serving as controls. The blank micelles exhibited no cytotoxicity against MCF-7 cells after 48 h of incubation. In contrast, while the DOX solution displayed significant cytotoxicity at all pH levels, the DOX-loaded micelles showed slightly lower cytotoxicity at neutral pH (pH 7.4). This reduced cytotoxicity is likely due to the sustained release characteristics of the DOX-loaded micelles and the differing cell uptake pathways between free DOX molecules and the micelles [230].

A novel platform of dual pH- and thermo-sensitive micelles based on poly(ethylene glycol)-b-poly(acrylamide-co-acrylonitrile-co-vinylimidazole) copolymer (mPEG-PAAV) has been recently developed for breast cancer therapy. In these micelles, the upper critical solution temperature (UCST) of the copolymer varies with different pH levels, allowing for controlled drug release. Two components, IR780, which serves as a near-infrared (NIR) absorber and effectively converts NIR laser energy into heat, and doxorubicin (DOX), were successfully encapsulated into the micelles. This combination enables NIR laser-controlled drug release and photoacoustic imaging (PAI) guidance for chemo-photothermal synergistic therapy. The results demonstrated that the IR780- and DOX-loaded mPEG-PAAV micelles, along with NIR laser irradiation, significantly improve the intracellular accumulation of DOX and induce breast cancer cell necrosis, apoptosis, and suppress migration in vitro. Moreover, the treatment with mPEG-PAAV micelles/IR780+DOX plus NIR laser irradiation eliminates the 4T1 breast tumor and thoroughly suppresses lung metastasis of breast cancer without observing any obvious adverse effects in mice. The results demonstrate that the hyperthermia-assisted chemotherapy using IR780- and DOX-loaded mPEG-PAAV micelles presents an effective combined treatment against subcutaneous and metastatic breast tumors [231].

## 5. Clinical Application of Polymer Micelles

A number of polymeric micellar formulations have been developed as drug delivery systems for cancer, demonstrating their therapeutic potential for cancer treatment. However, there are still some challenges regarding the clinical translation of these nanomedicines in practice, including their in vivo stability and drug retention, as well as ensuring a long circulation time in the bloodstream upon i.v. administration. These factors are important to ensure that the drug-loaded micelles reach the target site in the appropriate concentration in order to be effective. However, a limited number of polymeric micellar formulations are under or have completed clinical trials (Table 1). Some of them have already been approved in several countries and are currently being used in clinical practice (for example, Genexol™-PM, Nanoxel M^®^, and Zisheng^®^). It is important to note that most of these formulations are based on passive targeting of the drugs that are physically entrapped into core of the micelles through hydrophobic interactions between the core-forming block and of the drug and belong to the first-generation polymer micelles. Genexol-PM^®^, developed by Samyang Biopharmaceuticals Corporation in Seoul, South Korea, is one of the most clinically studied formulations based on PTX-loaded PEG-PDLL polymer micelles for treating various types of malignancies, including non-small cell lung cancer (NSCLC), metastatic breast cancer, ovarian cancer, and pancreatic cancer, as well as other types of cancer [67,68,232,233,234,235,236,237,238,239,240,241,242]. Phase II clinical trials have demonstrated that Genexol-PM is well-tolerated and exhibits significant antitumor activity when used as a monotherapy for metastatic breast cancer [238] and pancreatic cancer [242], reporting overall response rates (ORR) of 58.5% and 6.7%, respectively. When Genexol-PM is combined with cisplatin [234] or gemcitabine [67] for the treatment of NSCLC, the ORRs were 37.7% and 46.5%, respectively. This combination therapy also resulted in reduced myelotoxicity and emetogenicity compared to other treatments.

Zisheng^®^ (PM-Pac or PM-PTX) is a similar formulation based on PTX-loaded PEG-PDLL micelles, which was developed by Shanghai Yizhong Pharmaceutical Co., Ltd. (Shanghai, China), and was the first approved PTX-loaded PM in China. A phase 1 clinical study conducted on patients with advanced solid tumors revealed a high maximum tolerated dose (MTD) of 390 mg/m^2^ and promising antitumor activity without significant additional toxicity [243].

Nanoxel^®^-M is another formulation based on docetaxel (DTX)-loaded mPEG-b-PDLL micelles, developed by Samyang Biopharmaceutics Corporation (Seoul, Republic of Korea). To evaluate the safety and toxicity of Nanoxel^®^-M as an adjuvant therapy, either alone or in combination with cyclophosphamide, a multicenter trial was conducted in patients after surgery for early breast cancer. The results indicated that the micellar formulation decreased the incidence of taxane-induced peripheral neuropathy and thrombocytopenia compared to Taxotere^®^, and it did not cause hypersensitivity reactions [244].

Additionally, micellar formulations that contain doxorubicin (NK911) [245], paclitaxel (NK105) [71,72,246,247], SN-38 (NK012) [242,248,249,250,251,252], and cisplatin (NC-6004) [91,92,242,253,254,255] are currently under clinical evaluation (phase I or II). However, many of these formulations demonstrate moderate efficacy, despite improved tolerability.

SP1049C (Supratek Pharma) is a doxorubicin-loaded formulation based on Pluronic^®^ that shows significant single-agent efficacy in patients with adenocarcinoma of the esophagus and gastroesophageal junction (GEJ), along with an acceptable safety profile. A phase II clinical study involving 21 patients revealed an ORR of 47%. These results indicate that SP1049C has superior antitumor activity compared to standard formulations of doxorubicin [211,256,257].

The next-generation micellar formulations in clinical trials are based on chemical conjugation of the drug to the block copolymer through acid-labile pH-sensitive bonds, thus allowing longer retention time of the drug in the polymer micelles during circulation (NC 6300) or are based on active targeting using a specific ligand, thus ensuring effective targeting of the drug to tumor sites (BIND-014).

NC-6300 (NanoCarrier Co., Ltd., Kashiwa, Japan) is a nanoparticle formulation of epirubicin that has a pH-sensitive linker conjugated to epirubicin. A first-in-human, phase 1 study was conducted to evaluate the safety, tolerability, efficacy, and pharmacokinetics of NC-6300 administered as monotherapy in patients with advanced or recurrent solid tumors. The study reveals that NC-6300 was well-tolerated in patients with various solid tumors and exhibited less toxicity than conventional epirubicin formulations. The recommended phase 2 dose was set as 170 mg/m^2^, which was also the MTD [258]. A phase 1b, dose-escalation trial of NC-6300 monotherapy in patients with soft-tissue sarcoma (n = 17), one with osteosarcoma, and eleven with other solid tumors was conducted. The objective response rate in the evaluable population was 11%, as a partial response was observed in angiosarcoma and endometrial stromal sarcoma. The dose-limiting toxicities included thrombocytopenia, stomatitis, lung infection, and febrile neutropenia. The MTD and RD for phase 2 studies were determined to be 185 mg/m^2^ and 150 mg/m^2^, respectively. Based on these results, an expansion cohort was enrolled [242]. Patients received the recommended dose of 150 mg/m^2^ intravenously (IV) once every three weeks. The median progression-free survival (mPFS) for all subjects was 5.4 months (3.8 months for patients who had prior anthracycline treatment and 8.2 months for those without such treatment) [242]. The most common adverse events (AEs) included neutropenia, thrombocytopenia, leukopenia, anemia, fatigue, and nausea. These results demonstrated that NC-6300 is well-tolerated, showing promising activity in angiosarcoma patients without prior anthracycline treatment.

BIND-014 is a nanoparticle formulation containing docetaxel that targets the prostate-specific membrane antigen (PSMA). A first-in-human phase I trial was conducted to determine the safety, pharmacokinetics, and antitumor activity of BIND-014 in patients with advanced solid tumors [259]. The results demonstrated that BIND-014 was well-tolerated, with no unexpected toxicities. The most common drug-related toxicities (>20% of patients) included neutropenia, fatigue, anemia, alopecia, and diarrhea. BIND-014 demonstrated a dose-linear pharmacokinetic profile, which differs from that of docetaxel [259]. The recommended phase II dose of BIND-014 was 60 mg/m^2^ every three weeks or 40 mg/m^2^ weekly [259]. In a phase II clinical trial involving 42 patients with metastatic castration-resistant prostate cancer, a prostate-specific antigen (PSA) response was 30%, while a measurable disease response was 32%. Post-treatment results indicated that PSMA-positive CTCs were preferentially reduced. The following adverse events were predominantly grade 1 or 2 and included fatigue and nausea. Neuropathy occurs infrequently, and neutropenic fever was rare [260,261]. Additionally, phase II studies of BIND-014 were also conducted in patients with non-small cell lung cancer and squamous cell non-small cell lung cancer (NSCLC) [242].

**Table 1:** Polymer micelle formulations in clinical trials for cancer therapy (the information has been obtained from the national clinical trial website: https://clinicaltrials.gov).

Trade Name	Copolymer Type	Drug	Cancer Type	Phase of Clinical Trial	Identifier	Ref.
Genexol-PM^®^	mPEG-PDLLA	paclitaxel	hepatocellular carcinoma	phase II	NCT03008512	[242]
			epithelial ovarian cancer	phase I		[232]
			epithelial ovarian cancer	phase II		[233]
			ovarian cancer	phase I	NCT00877253	[242]
			ovarian cancer	phase I/II	NCT00886717	[242]
			non-small cell lung cancer	phase II		[234]
			non-small cell lung cancer	phase II	NCT01770795	[242]
			locally advanced head and neck squamous cell carcinoma	phase II	NCT01689194	[235]
			non-small cell lung cancer	phase II		[67]
			biliary tract cancer	phase II		[236]
			thymic epithelial tumors	phase II		[237]
			metastatic breast cancer	phase II		[238]
			metastatic breast cancer	phase II	NCT01784120	[242]
			metastatic breast cancer	phase II	NCT01169870	[242]
			urothelial cancer	phase II	NCT01426126	[239]
			adenocarcinoma of the pancreas	phase II	NCT02739633	[242]
			her2-negative breast cancer	phase III		[240]
			her2-negative metastatic breast cancer	phase II	NCT02263495	[241]
			metastatic breast cancer	phase III	NCT00876486	[242]
			pancreatic cancer	phase I/II	NCT00882973 NCT00111904	[242]
Nanoxel M^®^	PEG-b-PDLLA	Docetaxel-PM	breast cancer	phase IV		[244]
			advanced breast cancer	phase I	NCT00915369	[242]
			esophageal squamous cell carcinoma	phase II	NCT03585673	[242]
			head and neck squamous cell carcinoma	phase II	NCT02639858	[242]
			breast cancer	phase III	NCT05207514	[242]
			bladder cancer	phase III	NCT02982395	[242]
NK 911	PEG-b-P(Asp- DOX)	doxorubicin	pancreatic cancer	phase I		[245]
NK 105	mPEG-b- modified P(Asp)	paclitaxel	breast, gastric, esophageal, renal pelvis, prostate, and bladder tumors	phase I		[72]
			pancreatic, bile duct, gastric, and colon cancers	phase I		[71]
			advanced gastric cancer	phase II		[246]
			breast cancer	phase III		[262]
			breast cancer	phase III	NCT01644890	[247]
NK012	PEG-b-P(Glu)	SN-38 (active metabolite of irinotecan)	advanced solid tumors (lung, breast, ovarian, esophageal, gastric, colon, and endometrial cancers)	phase I		[248]
			solid tumors (colorectal, pancreatic, esophageal, and small and non-small cell lung cancers)	phase I		[249]
			gastrointestinal malignancies	phase I		[250]
			multiple myeloma	phase I/II		[251]
			colorectal cancer	phase II		[252]
			advanced solid tumors/metastatic colorectal cancer	phase I	NCT01238939	[242]
			advanced solid tumors/triple negative metastatic breast cancer	phase I	NCT01238952	[242]
			refractory solid tumors	phase I	NCT00542958	[242]
			small-cell lung cancer	phase II	NCT00951613	[242]
			triple negative breast cancer	phase II	NCT00951054	[242]
NC-6004	PEG-P(Glu)	cisplatin	advanced solid tumors (lung, colon, pancreatic, esophageal, and renal cancers)	phase I		[91]
			advanced solid tumors (carcinoma, neuroendocrine tumors)	phase I		[253]
			squamous cell carcinoma of the head and neck	phase I	NCT02817113	[242]
			advanced solid tumors or non-small cell lung, biliary tract, and bladder cancer	phase I b/II	NCT02240238	[254]
			squamous non-small cell lung carcinoma, biliary tract, and bladder cancers	phase II		[255]
			recurrent or metastatic squamous cell carcinoma of the head and neck	phase IIa/IIb	NCT03771820	[242]
			squamous cell carcinoma of the head and neck	phase II	NCT03109158	[242]
			locally advanced or metastatic pancreatic cancer	phase I/II	NCT00910741	[92]
			advanced or metastatic pancreatic cancer	phase III	NCT02043288	[242]
NC-6300	PEG-b-PASP	epirubicin	advanced solid tumors (urothelial, breast, and other cancers)	phase I		[258]
			sarcoma, osteosarcoma, and other tumors	phase Ib		[263]
			advanced solid tumors or advanced, metastatic, or unresectable soft-tissue sarcoma	phase Ib/II	NCT03168061	[242]
BIND-014	PEG-PLA	docetaxel	advanced solid tumors (lung, head and neck, ovarian, prostate, rectal, and other cancers)	phase I		[259]
			metastatic castration-resistant prostate cancer	phase II		[260]
			metastatic castration-resistant prostate cancer	phase II	NCT01812746	[261]
			KRAS mutation positive or squamous cell non-small cell lung cancer	phase II	NCT02283320	[242]
			urothelial carcinoma, cholangiocarcinoma, cervical cancer, and squamous cell carcinoma of the head and neck	phase II	NCT02479178	[242]
			advanced or metastatic cancer	phase I	NCT01300533	[242]
			non-small cell lung cancer	phase II	NCT01792479	[264]
SP1049C	Pluronic (R) L61/F127	doxorubicin	colorectal, esophageal, and lung cancer, soft-tissue sarcoma, mesothelioma, and other cancers	phase I		[256]
			esophageal adenocarcinoma	phase II		[211]
			adenocarcinoma of the esophagus and gastroesophageal junction	phase II		[257]
Zisheng^®^	mPEG-PDLLA	paclitaxel	advanced solid tumors	phase I	NCT04778839	[242,243]

## 6. Conclusions

In conclusion, the field of polymeric micelles for cancer therapy has developed from using conventional passive carriers to highly sophisticated smart delivery systems. Despite the extensive preclinical research presented, only a limited number of polymer micelle formulations have reached clinical approval. The main challenges include the low drug-loading capacity of polymer micelles, insufficient tumor accumulation, instability of polymer micelles in the bloodstream, and interaction with blood components like plasma proteins, which can lead to disintegration of the micelles and “burst release” of the drug before reaching the tumor site. Additionally, the effectiveness of passive targeting through the enhanced permeability and retention (EPR) effect can vary significantly depending on the tumor types, tumor progression, and diverse demographic groups. The interdisciplinary collaboration among polymer chemistry, biology, pharmaceutical sciences, and clinical research is expected to enhance the optimization, validation, and clinical adoption of polymer micelle-based nanomedicines. In addition, artificial intelligence (AI) can be used to optimize nanocarrier design and predict encapsulation efficiency and therapeutic efficacy, as well as drug release from long-acting injectable formulations. This can lead to more effective, targeted, and patient-specific therapies with controlled release profiles [265]. This progress aims to contribute to safer and more effective cancer treatments. Future research should focus on rational polymer design with simplified architecture as well as scalable and reproducible manufacturing processes. The establishment of well-defined structure-property and function relationships is crucial. There is also a need for greater emphasis on understanding the in vivo behavior of micelles, their stability under physiological conditions, tumor heterogeneity, and variability in enhanced permeability and retention (EPR) effects among patients. From a clinical perspective, the development of standardized evaluation protocols, predictive preclinical models, and biomarker-guided patient stratification will be essential to bridge the gap between laboratory success and therapeutic impact.

## Data Availability

No new data were created or analyzed in this study.

## Abbreviations

The following abbreviations are used in this manuscript:

EPR	Enhanced permeability and retention
mPEG	Methoxy-poly(ethylene glycol)
FRP	Free radical polymerization
ROP	Ring-opening polymerization
LRP	Living radical polymerization
CRO	Controlled radical polymerization
ATRP	Atom transfer radical polymerization
NMP	Nitroxide-mediated radical polymerization
RAFT	Reversible addition–fragmentation chain transfer polymerization
CMC	Critical micelle concentration determination
TEM	Transmission electron microscopy
cryoTEM	Cryo-transmission electron microscopy
DLS	Dynamic or quasi-elastic light scattering
AFM	Atomic force microscopy
SAXS	Small-angle X-ray scattering
SANS	small-angle neutron scattering
FRET	Förster Resonance Energy Transfer
PLA	Polylactide
PCL	Polycaprolactone
PLGA	Poly(lactic-co-glycolic acid)
RES	Reticuloendothelial system
DTX	Docetaxel
PTX	Paclitaxel
DOX	Doxorubicin
CDDP	Cisplatin
DACHPt	(1,2-diaminocyclohexane)platinum(II)
CPT	Camptothecin
CUR	Curcumin
9-NC	9-nitro-camptothecin
NSCLC	Non-small cell lung cancer
DLT	Dose-limiting toxicities
FA	Folic acid
FR	Folic receptors
DSPE	Distearoylphosphatidylethanolamine
Tf	Transferrin
BBB	Blood–brain barrier
TfRs	Transferrin receptors
mAb 2C5	Monoclonal antibody 2C5
EGFR	Epidermal Growth Factor Receptor
Apts	Aptamers
PSMA	Prostate-specific membrane antigen PSMA
Poly(His)	Poly(L-histidine)
PHSM	pH-responsive mixed micelles (PHSM)
PAE	Poly(β-amino ester)
TRITC	Tetramethylrhodamine isothiocyanate
Chol	Cholesterol
mM	Mixed micelles
SHA	Stearate-modified hyaluronic acid
PDPA	Poly(2-(diisopropylamino) ethyl methacrylate
TPGS	D-α-tocopheryl polyethylene glycol 1000 succinate
PAA	Poly(acrylic acid)
EGVE	Ethyl glycol vinyl ether
TPP	Triphenylphosphonium bromide
EPI	Epirubicin
TRPs	Thermosensitive polymers
LCST	Lower critical solution temperature
UCST	Upper critical solution temperature
PNIPAM	Poly(N-isopropylacrylamide)
AAm	Acrylamide
HMAAm	Hydroxymethylacrylamide
DMAAm	Dimethylacrylamide
PEO-PPO-PEO	Poly(ethylene oxide)-poly(propylene oxide)-poly(ethylene oxide)
MDR	Multidrug-resistant drug
ALN	Alendronate
GSH	Glutathione
αLA	Alpha-lipoic acid
CS	Chondroitin sulfate
ROS	Reactive oxygen species
